# Polydopamine and eumelanin molecular structures investigated with *ab initio* calculations†
†Electronic supplementary information (ESI) available. See DOI: 10.1039/c6sc04692d



**DOI:** 10.1039/c6sc04692d

**Published:** 2016-11-02

**Authors:** Chun-Teh Chen, Francisco J. Martin-Martinez, Gang Seob Jung, Markus J. Buehler

**Affiliations:** a Laboratory for Atomistic and Molecular Mechanics (LAMM) , Department of Civil and Environmental Engineering , Massachusetts Institute of Technology , 77 Massachusetts Ave. , Cambridge , Massachusetts 02139 , USA . Email: mbuehler@mit.edu

## Abstract

A set of computational methods that contains a brute-force algorithmic generation of chemical isomers, molecular dynamics (MD) simulations, and density functional theory (DFT) calculations is reported and applied to investigate nearly 3000 probable molecular structures of polydopamine (PDA) and eumelanin. All probable early-polymerized 5,6-dihydroxyindole (DHI) oligomers, ranging from dimers to tetramers, have been systematically analyzed to find the most stable geometry connections as well as to propose a set of molecular models that represents the chemically diverse nature of PDA and eumelanin. Our results indicate that more planar oligomers have a tendency to be more stable. This finding is in good agreement with recent experimental observations, which suggested that PDA and eumelanin are composed of nearly planar oligomers that appear to be stacked together *via* π–π interactions to form graphite-like layered aggregates. We also show that there is a group of tetramers notably more stable than the others, implying that even though there is an inherent chemical diversity in PDA and eumelanin, the molecular structures of the majority of the species are quite repetitive. Our results also suggest that larger oligomers are less likely to form. This observation is also consistent with experimental measurements, supporting the existence of small oligomers instead of large polymers as main components of PDA and eumelanin. In summary, this work brings an insight into the controversial structure of PDA and eumelanin, explaining some of the most important structural features, and providing a set of molecular models for more accurate modeling of eumelanin-like materials.

## Abstract 2

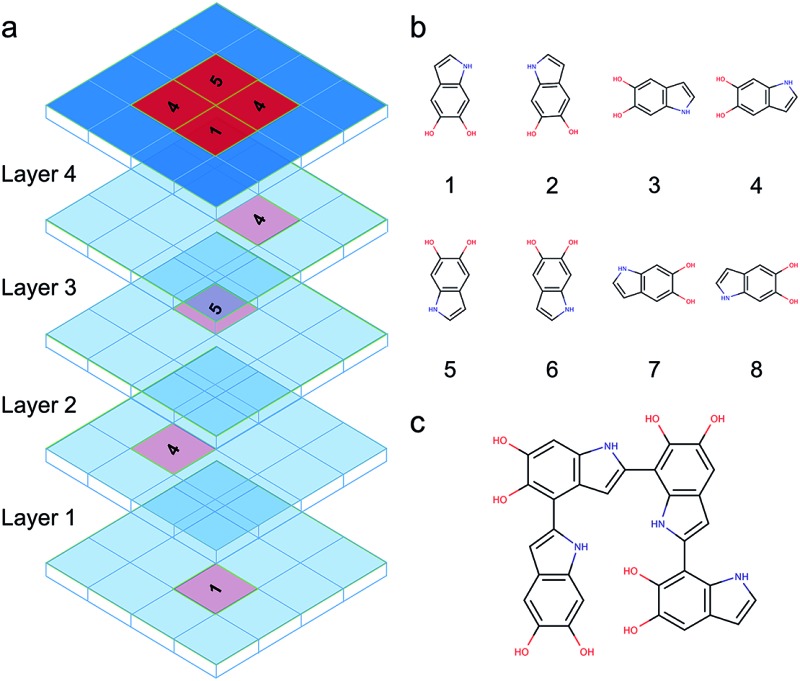
A set of computational methods that contains a brute-force algorithmic generation of chemical isomers, molecular dynamics (MD) simulations, and density functional theory (DFT) calculations is reported and applied to investigate nearly 3000 probable molecular structures of polydopamine (PDA) and eumelanin.

## Introduction

Polydopamine (PDA), first reported by Messersmith, Lee, and co-workers in 2007,^1^ is a black insoluble material produced by the oxidative polymerization of dopamine under alkaline conditions. PDA shares chemical functionalities (*i.e.*, catechol and nitrogen-containing groups) with mussel adhesive proteins such as *Mytilus edulis* foot protein-5 (Mefp-5).^2^ Mussels can attach to various types of surfaces with high bonding strength, even under harsh marine conditions. Such extraordinarily robust adhesion is due to the presence of significant amounts of catechol and amine-rich amino acids in their threads.^3–5^ Accordingly, synthetic materials containing these functional groups are the object of growing interest, not only for adhesives but also for many other applications. PDA is a mussel-inspired material since its fundamental building blocks contain both catechol and amine groups. As with mussel threads, PDA can spontaneously deposit on the surface of almost any material regardless of its chemical nature. Therefore, PDA has drawn extensive interest as a versatile surface functionalization and coating material for a broad range of applications.^1,6,7^ Surface coating provides protection of the underlying material against external erosion by agents such as strong oxidants and acids. Furthermore, surface modification can change the surface properties and create additional functionalities of the underlying material. Due to the advantages mentioned above, PDA has entered the scene of materials science in recent years, with applications that go beyond surface coatings,^8,9^ into the fields of solar energy,^10^ water purification,^11^ shape memory polymer,^12^ microrobots,^13^ biomedicine,^14–16^ and nanotechnology.^9,17^

On the other hand, eumelanin, the most common type of melanin, is a natural pigment in the human skin, hair, and eyes. Eumelanin shares many physiochemical properties with PDA due to their similarities in structures and chemical functionalities. Eumelanin is an essential pigment in most organisms and has been studied for decades. X-ray diffraction studies,^18,19^ scanning tunneling microscopy (STM) measurements,^20–22^ atomic force microscopy (AFM) and transmission electron microscopy (TEM) images^23,24^ suggested the existence of small nearly planar oligomers that appear to be stacked together *via* π–π interactions to form graphite-like layered aggregates. However, proper explanations for the small molecular size of these oligomers and their detailed molecular structures have not been provided yet. As a result, unlike the vast majority of natural pigments (*e.g.*, chlorophyll and carotenoid), eumelanin cannot be described in terms of a well-defined structure, but rather as a polydisperse mixture of oligomers. The structural investigations of eumelanin are challenging due to its amorphous characteristic as well as insolubility in water and most organic solvents. Therefore, it is currently impossible to provide an accurate picture of eumelanin structure beyond a description of its fundamental building blocks, which are 5,6-dihydroxyindolequinone (DHI) and 5,6-dihydroxyindole-2-carboxylic acid (DHICA).^25,26^ In addition, very little information is available regarding the further evolution of these building blocks to eumelanin structure, and its polymerization mechanism is also ambiguous.

The lack of precise knowledge on the molecular structures and polymerization mechanism of eumelanin does not stop the applicability of synthetic eumelanin-like materials, among which PDA is the most popular one. Dopamine, the main constituent of PDA, stands as a natural choice for synthesizing universal coating materials since it is the simplest building block containing the required catechol and amine groups. DHI, the intermediate oxidized product of dopamine, is the precursor of PDA and has been successfully synthesized and characterized in experiments. Consequently, the fundamental building blocks of PDA are DHI and its redox forms, as well as a portion of uncyclized amine-containing units. A recent study suggested that increasing the dopamine concentration while synthesizing PDA leads to higher proportions of uncyclized amine-containing units. On the other hand, lower dopamine concentrations lead to higher levels of DHI.^27^ However, unlike eumelanin, DHICA-derived units are absent as building blocks in PDA.^28^ As a result, even though PDA is sometimes called synthetic eumelanin in literature,^29^ these two materials are not synonymous. In this work, to simplify the problem, we ignore the differences between PDA and eumelanin and consider them as similar materials composed of DHI and its redox forms. As with eumelanin,^30^ despite the significant effort on structural investigations of PDA since it was first reported in 2007, the structure of PDA has yet to be determined. Recently, an experimental study^29^ using solid state spectroscopic techniques suggested that PDA is a supramolecular aggregate of DHI monomers, which are held together by a combination of charge transfer, π-stacking, and hydrogen bonding interactions. Another experimental study^31^ using high-performance liquid chromatography-mass spectrometry (HPLC-MS) reported that a physical, self-assembled trimer of (dopamine)_2_/DHI exists in PDA. However, it is generally accepted in this field that the polymerization of DHI generates a group of covalent oligomers including dimers, trimers, tetramers, and even larger oligomers. From the chemical analysis, it is clear that the existence of various redox forms of DHI monomers, but the way such monomers connect together to form oligomers is still unclear due to the formation of numerous intermediates during the polymerization. This variety of oligomers and redox forms that constitute PDA and eumelanin is referred to as chemical disorder model.^32^

The absent of proper molecular models for PDA and eumelanin implies a fundamental drawback that hampers a better understating, fast development, and property optimization of these materials. Although the science and applications of PDA-based materials have rapidly advanced in recent years, most studies in this field were based on empirical approaches instead of a solid framework of structure–function relationships due to this structural controversy. In this work, a set of computational methods, which includes a brute-force algorithmic generation of chemical isomers, molecular dynamics (MD) simulations, and density functional theory (DFT) calculations, is implemented to investigate molecular structures of PDA and eumelanin. The investigation begins with analyzing the reactivity of DHI and its related radical species that lead to the formation of PDA and eumelanin. The most reactive positions of DHI are identified, and a possible polymerization mechanism of DHI in different redox forms based on spin density calculations, is also provided. In addition, early studies showed that the size of eumelanin protomolecules is around 15–20 Å, suggesting that tetramers and pentamers formed by covalently bonded DHI monomers are the most probable molecular sizes of eumelanin.^18–22^ A recent study based on mass spectroscopy results of DHI-melanin also suggested that the majority constituents of the material are tetramers and pentamers of DHI.^32^ Consequently, we narrow down our investigation to DHI oligomers up to tetramers. Within the framework of this bottom-up approach and considering the location of the most reactive positions of DHI, we systematically generate and analyze all probable early-polymerized DHI oligomers, ranging from dimers to tetramers, to provide a fundamental explanation of some important structural features of PDA and eumelanin, as well as to propose a set of molecular models that represents the chemically diverse nature of these materials.

## Results and discussion

### DHI radical polymerization

**Fig. 1:**
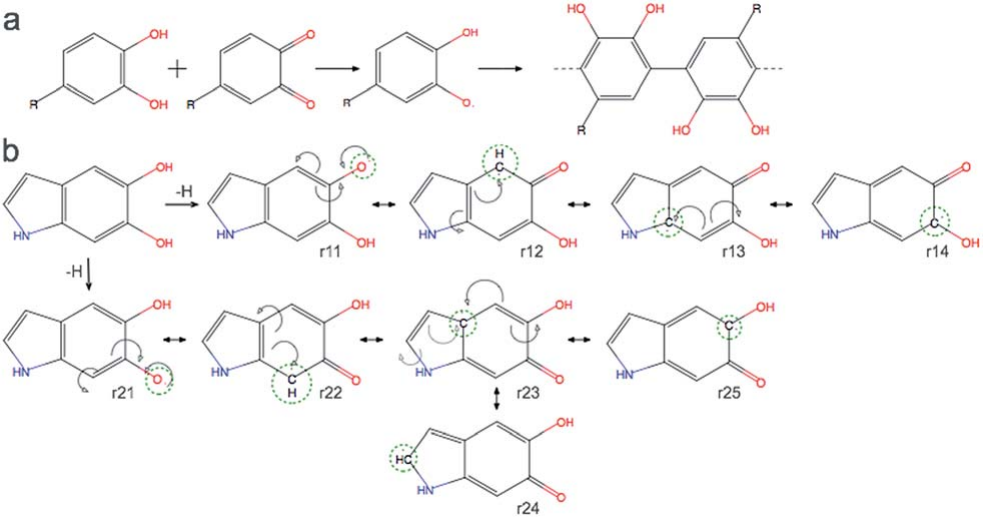
(a) Simplified possible reaction mechanism for catechol polymerization, based on literature data. Possible pathway for PDA polymerization *via* reverse dismutation and aryloxy coupling, adapted and modified from ref. 33. (b) Two possible DHI radical species and the resonant structures resulting from the delocalization of the radical electron.

**Fig. 2:**
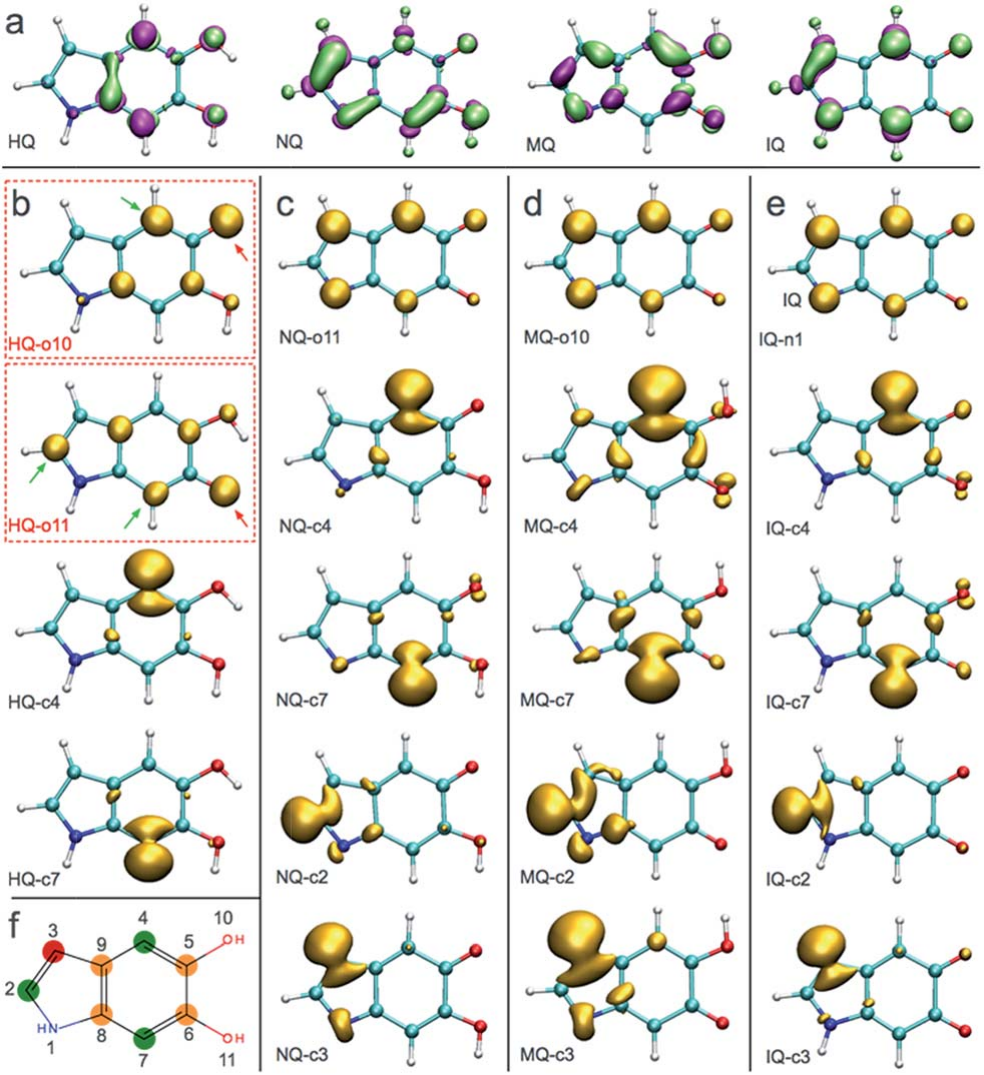
Reactivity of DHI (HQ) and the spin densities of its radical species. The Fukui function and spin densities for different 2-electron oxidation products of DHI (HQ), namely HQ (non-oxidized), NQ, MQ, and IQ, and possible sites of polymerization. In the Fukui function, green volumes indicate regions that are suitable to undergo electrophilic attack and purple ones indicate regions that are suitable to undergo nucleophilic attack. In the spin density plots, gold indicates regions where the unpaired electron is delocalized. (a) Dual descriptor of the Fukui function for all 2-electron oxidation products of DHI (HQ). (b) Spin densities of the possible radical species derived from HQ. Red dashed box indicates the two main radicals generated by the accepted reaction mechanism. (c) Spin densities of the possible radical species derived from NQ (d) spin densities of the possible radical species derived from MQ. (e) Spin densities of the possible radical species derived from IQ. (f) Summary of possible atomic positions for polymerization.

A detailed investigation of the reaction mechanisms of DHI that lead to the formation of DHI oligomers, including a complete description of potential energy surfaces, activation energies, and reaction pathways, goes beyond the scope of this work. Despite such complexity, we still can analyze the reactivity of DHI to form radical species within the context of some accepted mechanisms of catechol polymerization, as well as describe the spin density and radical resonant structures that lead to the expected connections between DHI monomers. Therefore, to support the prevalence of certain atomic connections in the formation of DHI oligomers, and to provide some insight into the polymerization process that leads to the molecular structures of eumelanin-like materials, DFT calculations are performed for different redox forms of DHI. Fig. 1a summarizes a possible mechanism for catechol polymerization based on literature data.^33–35^ In this mechanism, *o*-quinone groups react *via* aryloxy radical coupling with the unoxidized catechol to form crosslinks. This reaction scheme can be extrapolated to the case of PDA and eumelanin formation, for which the catechol functional group is thought to be the main responsible of the polymerization. Fig. 1b shows two possible DHI radical species involved in these reactions, and the resonant structures resulting from the delocalization of the radical electron. The dashed circular lines in green highlight the atomic positions where the unpaired electron would delocalize in different resonant structures. Calculating the reactivity of the molecules that lead to these radicals, as well as the spin density of these radical species, is more accurate than depicting all possible resonant structures for different tautomers. Quantum chemical calculations are required to describe the electronic effects involved in the reaction. Here DFT calculations are implemented to compute the so-called Fukui function and spin density of the radical species, from monomers to tetramers. The Fukui function gives an indication of the formation of the radicals while the spin density shows the delocalization of the unpaired electrons that control the radical reaction. Fig. 2a shows the dual descriptor of the Fukui function for DHI and its redox forms. For convenience and consistency with the other species discussed, the reduced form of DHI is denoted as HQ, and the oxidized forms are denoted as quinone-imine (NQ), quinone-methide (MQ) and indolequinone (IQ). Accordingly, four different monomers are considered, namely HQ, NQ, MQ and IQ, where the colored surfaces represent those molecular regions suitable for undergoing either nucleophilic (purple) or electrophilic (green) attack. The results indicate that the reactivity of DHI is mainly concentrated in the catechol functional group, which makes the reaction scheme shown in Fig. 1a a possible reaction scheme for DHI polymerization. Despite the uncertainty in the actual mechanism of the radical generation, an eventual electrophilic attack over the oxygen positions of DHI would be responsible for the formation of the subsequent radical species in the oxygen atoms, while a nucleophilic attack over the carbon positions of the catechol ring would form the subsequent carbon radicals. We are aware that other mechanisms could also be possible, and we only emphasize the localization of the reactivity in the atomic positions consistent with the proposed models. According to this, there are four possible radical species that can be formed in the catechol functional group, coming from hydrogen abstraction over oxygen atoms (atoms 10 and 11 in Fig. 2f) and over the two external carbon atoms (atoms 4 and 7 in Fig. 2f).

Fig. 1b shows the spin density of these four HQ radical species, where the yellow surfaces indicate the probability density for the unpaired radical electron. We name the radicals where a hydrogen atom is removed from one of the hydroxyl functional groups HQ-o10 and HQ-o11, while HQ-c4 and HQ-c7 are the species where the unpaired electron is generated in one of the carbon atoms of the catechol ring. HQ-o10 and HQ-o11 are highlighted in a red box since these are the species that would initially form, in agreement with reaction scheme shown in Fig. 1a, and thus they might be responsible for the initiation of the radical reaction. From Fig. 2b, it is clear that the atomic positions with higher spin density coincide with those suggested in Fig. 1b by studying the resonant structures. The result shows the potential of using spin density analysis for quickly exploring these radicals. Within this context, HQ-o10 and HQ-o11 would potentially attack HQ, NQ, MQ and IQ to form more radical species through hydrogen abstraction mechanisms, thus generating all radicals shown in Fig. 2c–e. Those species in which the radical is generated by hydrogen abstraction in the hydroxyl group present higher delocalization of the unpaired electron, which is located not only in that oxygen atom but also in some of the carbon atoms. On the contrary, when the radical species are generated in a carbon atom of the catechol, the unpaired electron remains mainly localized in that carbon atom.

Fig. 2f summarizes the atomic positions that are most likely to polymerize based on the spin density analysis of all possible radical species. The position highlighted in red corresponds to the carbon atom in which the spin density is lower among all different structures, and thus the unpaired electron in this carbon atom is less likely to appear. Accordingly, we expect less number of crosslinking through this position. The positions highlighted in orange (5-, 6-, 8-, and 9-position) present localization of the spin density, but these positions are more difficult to access due to steric effects. On the contrary, the positions highlighted in green (2-, 4-, and 7-position) are easy to access during polymerization and also present higher spin density in most of the cases. Most importantly, in the main radical species generated from HQ, namely HQ-o10 and HQ-o11, these three positions are indeed in agreement with the most probable connections in DHI oligomers. Even though there are four carbon atoms in DHI that are suitable for polymerization, only these three are likely to polymerize in the radical reaction, as we will discuss later. Based on the polymerization sites described for the monomers, we perform a similar analysis for dimer and trimers.

**Fig. 3:**
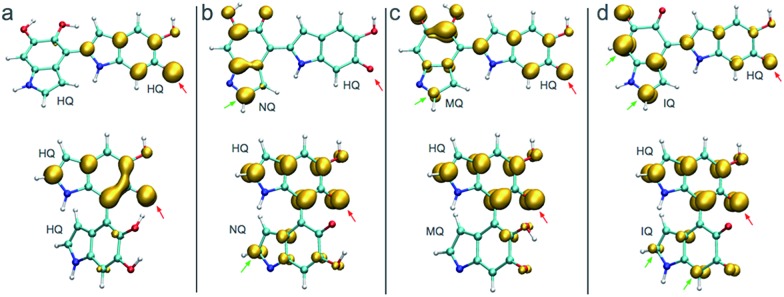
Spin densities of various trimers and tetramers in different redox forms. Red arrows indicate where the hydrogen abstraction has taken place to produce the radical. Green arrows indicate high spin density regions that enable further polymerization. (a) Spin densities of two possible radicals for HQ–HQ dimer. (c) Spin densities of two possible radicals for HQ–MQ dimer. (d) Spin densities of two possible radicals for HQ–IQ dimer.

Fig. 3 shows the spin density of some HQ–HQ, HQ–NQ, HQ–MQ and HQ–IQ radical dimers that would be generated from the reaction with HQ-o10 and HQ-o11. The red arrows indicate the position where the hydrogen abstraction occurs, and the green arrows indicate the positions suitable for further functionalization based on the spin density. It is important to remark that HQ–HQ radical presents the spin density mainly localized in the monomer where the hydrogen abstraction occurs. On the contrary, HQ–NQ and HQ–IQ radical dimers present spin density localization in the atomic positions away from the initial radical, and also consistent with the selectivity for 2-, 4-, and 7-position polymerization. This is an important result for the fundamental understanding of the polymerization mechanism of DHI since it supports the need of having oxidized species of DHI in the reaction media. To further support this result, we extend the spin density analysis to larger oligomers.

**Fig. 4:**
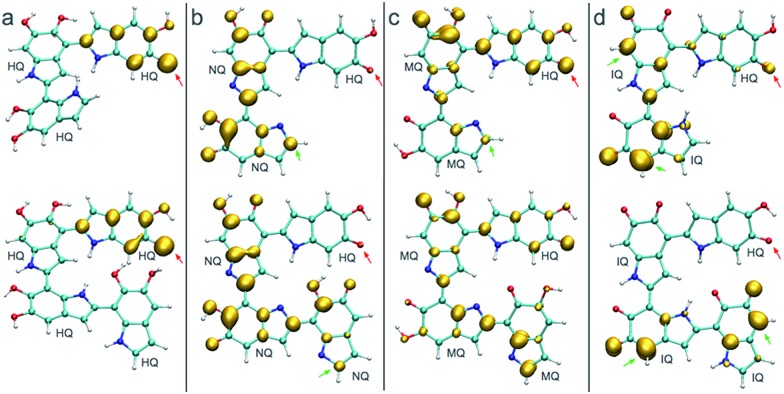
Spin densities of different trimers and tetramers at different redox forms. Red arrows indicate where the hydrogen abstraction has taken place to produce the radical. (a) Spin densities of possible radicals in a HQ polymerization. (b) Spin densities of possible radicals in a HQ–NQ polymerization. (c) Spin densities of possible radicals in a HQ–MQ polymerization. (d) Spin densities of possible radicals for HQ–IQ polymerization.

Fig. 4 shows the spin density of some radical trimers and tetramers. As with the previous figure, the red arrows indicate the positions where the hydrogen abstraction occurs, and the green arrows indicate the positions suitable for further functionalization based on the spin density. The results are consistent with the previous analysis for dimers (Fig. 3), and supporting the idea of having oxidized species of DHI in the reaction media. In fact, when the radicals include NQ, MQ, or IQ species, the unpaired electron is delocalized all over the structure, as shown in the spin density plots, and would keep polymerizing through 2-, 4-, and 7-position.

### Proposed molecular structures for dimers

Based on the results discussed in the previous section, the unpaired electron in the radical species, a key player in the polymerization mechanism of DHI, is mainly localized in 2-, 4-, and 7-position. The result implies that the covalent bonds connecting DHI monomers to generate DHI oligomers are more likely to form at these positions. As a result, there are 6 structural unique dimers (constitutional isomers), which are made through 2,2′-, 2,4′-, 2,7′-, 4,4′-, 4,7′-, and 7,7′-position. Nevertheless, to evaluate the stability of all possible connectivity, a total of 18 dimers are generated using a brute-force algorithmic generator (see Computational details section). Fig. S1† shows the molecular structures of these 18 dimers. Note that this group of dimers not only covers all 6 structural unique dimers but also includes 12 redundant dimers with different conformations to find the most stable conformation of each structural unique dimer. Table S1† shows the relative energies compared to the most stable dimer as well as the covalent bonding positions of these 18 dimers. The Becke three-parameter Lee–Yang–Parr (B3LYP)^36,37^ functional is adopted for optimizing the molecular structures of these 18 dimers, together with the def2-QZVP^38^ basis set (see Computational details section). After discarding higher energy conformations, the most stable dimer is made through 2,2′-position, followed by 2,4′-position (+1.82 kcal mol^–1^), 4,4′-position (+2.19 kcal mol^–1^), 4,7′-position (+2.31 kcal mol^–1^), 2,7′-position (+2.82 kcal mol^–1^), and 7,7′-position (+3.20 kcal mol^–1^).

**Fig. 5:**
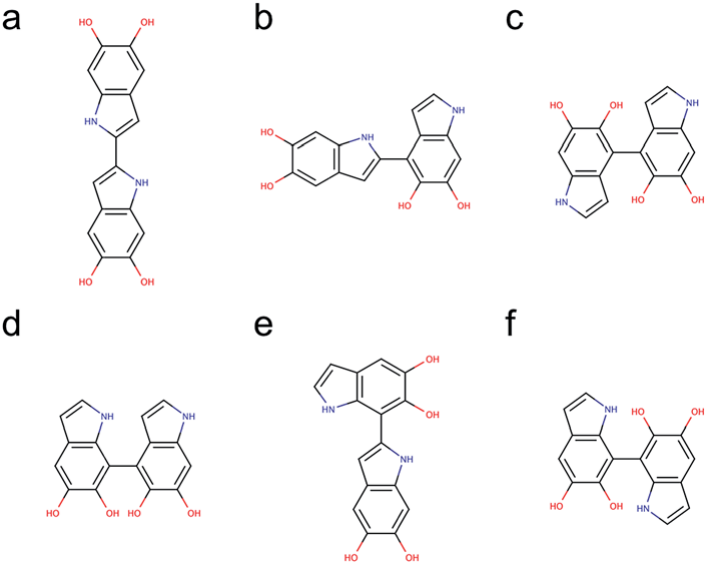
Molecular structures of 6 structural unique dimers. The most stable dimer is made through (a) 2,2′-position, followed by (b) 2,4′-position (+1.82 kcal mol^–1^), (c) 4,4′-position (+2.19 kcal mol^–1^), (d) 4,7′-position (+2.31 kcal mol^–1^), (e) 2,7′-position (+2.82 kcal mol^–1^), and (f) 7,7′-position (+3.20 kcal mol^–1^).

Fig. 5 shows the molecular structures of the 6 structural unique dimers in the order of their relative energies compared to the most stable one. The dimers made through 2,2′-position (Fig. 5a), 2,4′-position (Fig. 5b), and 2,7′-position (Fig. 5e), have been identified in experiments by oxidation of DHI.^39,40^ In addition, the dimer made through 4,7′-position (Fig. 5d) has also been verified in experiments.^41^ Although the dimers made through 4,4′-position (Fig. 5c) and 7,7′-position (Fig. 5f) have not been isolated in experiments, these two covalent bonding formations have been identified by oxidation of DHICA.^42^ Due to the structural similarity between DHI and DHICA, we expect that these two covalent bonding formations can also form in oxidation of DHI. As a result, the dimers shown in Fig. 5 are all likely to exist in PDA and eumelanin. The activation energies for DHI monomers to form various DHI oligomers are hard to calculate without knowing the comprehensive polymerization mechanism. However, the equal chemical functionalities and similar structural conformations between the different species involved in the polymerization suggest comparable energy barriers be expected. Furthermore, it has been experimentally proven that the polymerization of DHI occurs. Hence, it is reasonable to assume that the formation of oligomers is kinetically possible in most of the cases. Consequently, we assume that the energy of an oligomer determines its prevalence with respect to others. These assumptions define the theoretical framework in this work for evaluating different oligomers that contribute to the overall chemical structure of PDA and eumelanin.

### Proposed molecular structures for trimers

**Fig. 6:**
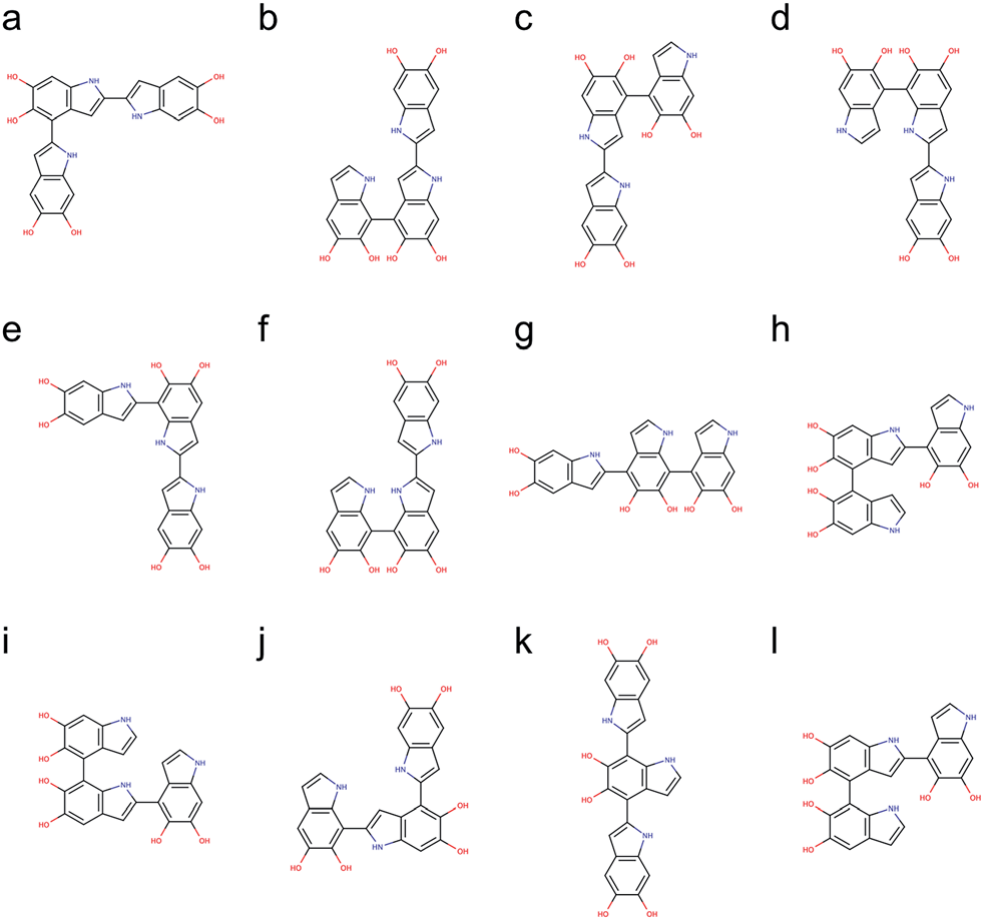
Molecular structures of the top 12 stable trimers. Those trimers are made through (a) 2,4′- & 2,2′-position, (b) 7,4′- & 2,2′-position (+0.78 kcal mol^–1^), (c) 2,2′- & 4,4′-position (+0.98 kcal mol^–1^), (d) 2,2′- & 7,4′-position (+1.08 kcal mol^–1^), (e) 2,2′- & 7,2′-position (+1.43 kcal mol^–1^), (f) 7,7′- & 2,2′-position (+2.31 kcal mol^–1^), (g) 2,4′- & 7,4′-position (+2.41 kcal mol^–1^), (h) 4,2′- & 4,4′-position (+2.51 kcal mol^–1^), (i) 4,2′- & 7,4′-position (+2.64 kcal mol^–1^), (j) 7,2′- & 4,2′-position (+2.65 kcal mol^–1^), (k) 2,4′- & 7,2′-position (+2.71 kcal mol^–1^), and (l) 4,2′- & 4,7′-position (+2.73 kcal mol^–1^).

Following the same strategy applied for dimers, a total of 216 trimers are generated using the brute-force algorithmic generator. Fig. S2† shows the molecular structures of these 216 trimers. This group of trimers covers all 27 structurally unique trimers and also includes 189 redundant trimers with different conformations to find the most stable conformation of each structural unique trimer. Note that the B3LYP functional with the def2-QZVP basis set adopted for optimizing the molecular structures of the dimers is too expensive for the trimers. Consequently, the Becke–Lee–Yang–Parr (BLYP)^43,44^ functional is adopted for optimizing the molecular structures of the trimers, together with the def2-SVP^38^ basis set (see Computational details section). After discarding those structures with higher energy conformations, the top 12 stable trimers are identified, which are made through 2,4′- & 2,2′-position, 7,4′- & 2,2′-position (+0.78 kcal mol^–1^), 2,2′- & 4,4′-position (+0.98 kcal mol^–1^), 2,2′- & 7,4′-position (+1.08 kcal mol^–1^), 2,2′- & 7,2′-position (+1.43 kcal mol^–1^), 7,7′- & 2,2′-position (+2.31 kcal mol^–1^), 2,4′- & 7,4′-position (+2.41 kcal mol^–1^), 4,2′- & 4,4′-position (+2.51 kcal mol^–1^), 4,2′- & 7,4′-position (+2.64 kcal mol^–1^), 7,2′- & 4,2′-position (+2.65 kcal mol^–1^), 2,4′- & 7,2′-position (+2.71 kcal mol^–1^), and 4,2′- & 4,7′-position (+2.73 kcal mol^–1^). Fig. 6 shows the molecular structures of these top 12 stable trimers in the order of their relative energies. Note that the DFT calculations for the trimers are implemented using the small def2-SVP basic set instead of the large def2-QZVP basic set due to computational limitations. The basic set error in the calculations could be larger than the energy differences between the trimers (see Computational details section), and thus the rank in Fig. 6 might not be exactly correct. However, the group of trimers shown in Fig. 6 should be at least more stable than the other trimers. Consequently, instead of providing the exact rank for the trimers, here we can only propose a group of trimers that is more energetic favorable. In fact, the trimer made through 2,4′- & 2,7′-position (Fig. 6j) has been identified by oxidation of DHI.^39,40^

### Proposed molecular structures for tetramers

**Fig. 7:**
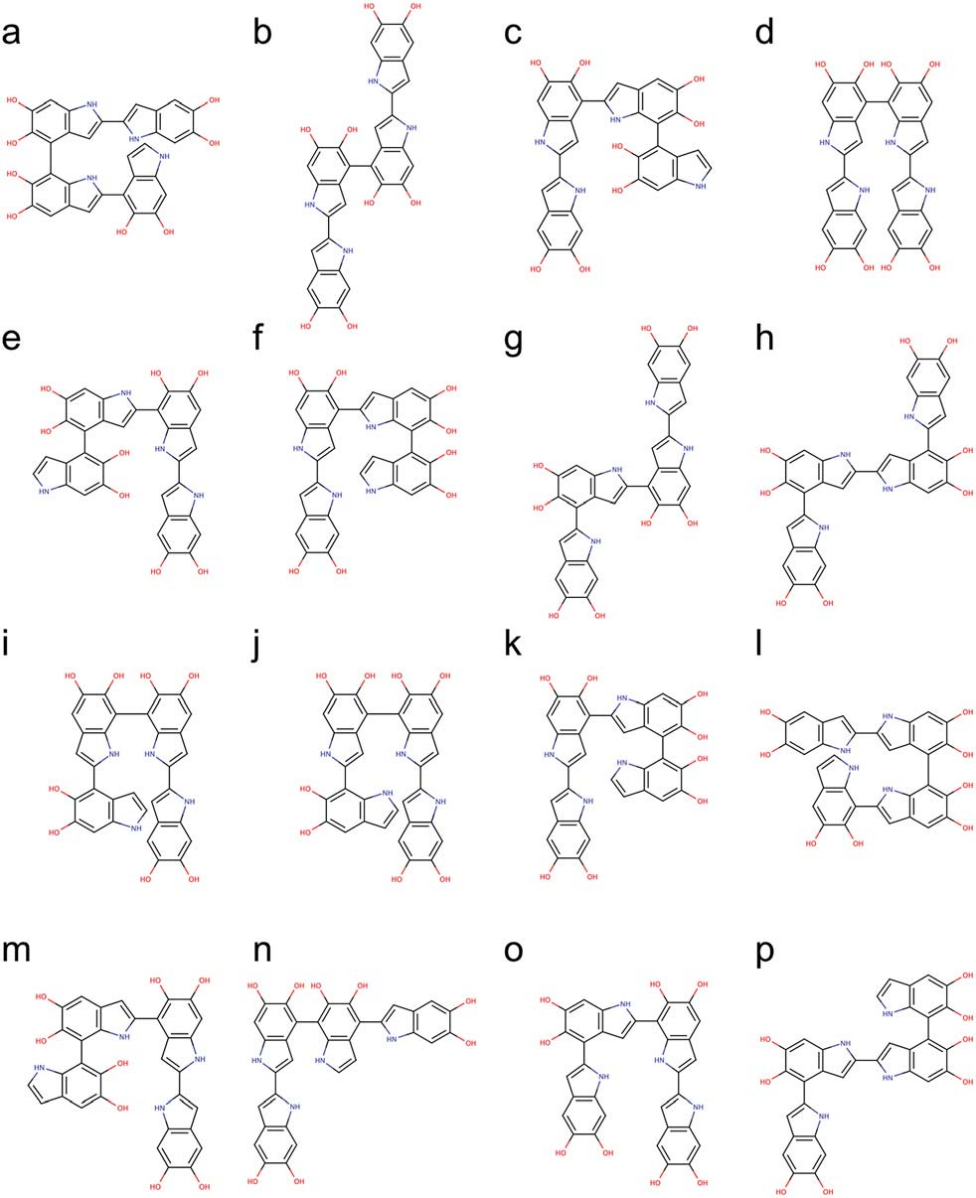
Molecular structures of the top 16 stable tetramers. Those tetramers are made through (a) 4,2′- & 7,4′- & 2,2′-position, (b) 2,2′- & 4,4′- & 2,2′-position (+0.59 kcal mol^–1^), (c) 2,2′- & 4,2′- & 4,4′-position (+1.03 kcal mol^–1^), (d) 2,2′- & 4,7′- & 2,2′-position (+1.08 kcal mol^–1^), (e) 2,2′- & 7,2′- & 4,4′-position (+1.18 kcal mol^–1^), (f) 2,2′- & 4,2′- & 7,4′-position (+1.20 kcal mol^–1^), (g) 2,4′- & 2,4′- & 2,2′-position (+1.33 kcal mol^–1^), (h) 2,4′- & 2,2′- & 4,2′-position (+1.50 kcal mol^–1^), (i) 2,2′- & 7,7′- & 2,4′-position (+1.56 kcal mol^–1^), (j) 2,2′- & 7,4′- & 2,7′-position (+1.91 kcal mol^–1^), (k) 2,2′- & 4,2′- & 4,7′-position (+2.03 kcal mol^–1^), (l) 7,2′- & 7,4′- & 2,2′-position (+2.06 kcal mol^–1^), (m) 2,2′- & 4,2′- & 7,7′-position (+2.11 kcal mol^–1^), (n) 2,2′- & 4,7′- & 4,2′-position (+2.22 kcal mol^–1^), (o) 2,4′- & 2,7′- & 2,2′-position (+2.24 kcal mol^–1^), and (p) 2,4′- & 2,2′- & 4,7′-position (+2.33 kcal mol^–1^).

The number of DHI oligomers that need to be investigated increases exponentially as the size increases. A total of 2592 tetramers are generated using the brute-force algorithmic generator. This group of tetramers covers all 162 structural unique tetramers as well as 2430 redundant tetramers that account for different conformations to find the most stable conformation of each structural unique tetramer. As with the calculations for the trimers, the BLYP^43,44^ functional is adopted for optimizing the molecular structures of the tetramers, together with the def2-SVP^38^ basis (see Computational details section). After removing the results of higher energy conformations, the systematic search for the most stable molecular structures of the tetramers is completed. Following the same strategy, the top 16 stable tetramers are identified, which are made through 4,2′- & 7,4′- & 2,2′-position, 2,2′- & 4,4′- & 2,2′-position (+0.59 kcal mol^–1^), 2,2′- & 4,2′- & 4,4′-position (+1.03 kcal mol^–1^), 2,2′- & 4,7′- & 2,2′-position (+1.08 kcal mol^–1^), 2,2′- & 7,2′- & 4,4′-position (+1.18 kcal mol^–1^), 2,2′- & 4,2′- & 7,4′-position (+1.20 kcal mol^–1^), 2,4′- & 2,4′- & 2,2′-position (+1.33 kcal mol^–1^), 2,4′- & 2,2′- & 4,2′-position (+1.50 kcal mol^–1^), 2,2′- & 7,7′- & 2,4′-position (+1.56 kcal mol^–1^), 2,2′- & 7,4′- & 2,7′-position (+1.91 kcal mol^–1^), 2,2′- & 4,2′- & 4,7′-position (+2.03 kcal mol^–1^), 7,2′- & 7,4′- & 2,2′-position (+2.06 kcal mol^–1^), 2,2′- & 4,2′- & 7,7′-position (+2.11 kcal mol^–1^), 2,2′- & 4,7′- & 4,2′-position (+2.22 kcal mol^–1^), 2,4′- & 2,7′- & 2,2′-position (+2.24 kcal mol^–1^), and 2,4′- & 2,2′- & 4,7′-position (+2.33 kcal mol^–1^). Fig. 7 shows the molecular structures of these top 16 stable tetramers in the order of their relative energies. As discussed before, the rank shown in Fig. 7 might not be exactly correct due to the accuracy in the calculations. However, the group of tetramers shown in Fig. 7 should be at least more energetic favorable than the other tetramers.

We are aware of the evidence of porphyrin-like tetramers that has been found in natural eumelanin pigment as well as in PDA by using electrochemical fingerprinting.^45^ These tetramers are made through 2,7′- & 2,7′- & 2,7′-position, creating an interior ring where all the nitrogen atoms are in the center, in an arrangement similar to porphyrin. Such arrangement was first proposed by Kaxiras^25,26^ and have been adopted to study the structural, mechanical, and optical properties of PDA and eumelanin in our previous computational work.^46,47^ However, this particular kind of molecular structures, in which an additional covalent bond is formed between the first and fourth monomers, is not considered in this work. The reason is that two hydrogen atoms need to be removed when an additional covalent bond. Therefore, the number of atoms in porphyrin-like tetramers is different from that in the tetramers considered in this work. Consequently, the energy comparison between tetramers with different numbers of atoms will be problematic. Despite PDA and eumelanin might contain porphyrin-like tetramers in certain concentrations, it is impractical to assume all tetramers are in the porphyrin-like arrangement. Therefore, considering other species of probable molecular structures that represent the chemically diverse nature of these materials, is important to perform more accurate simulations.

### Statistical characteristics of data for trimers and tetramers

**Fig. 8:**
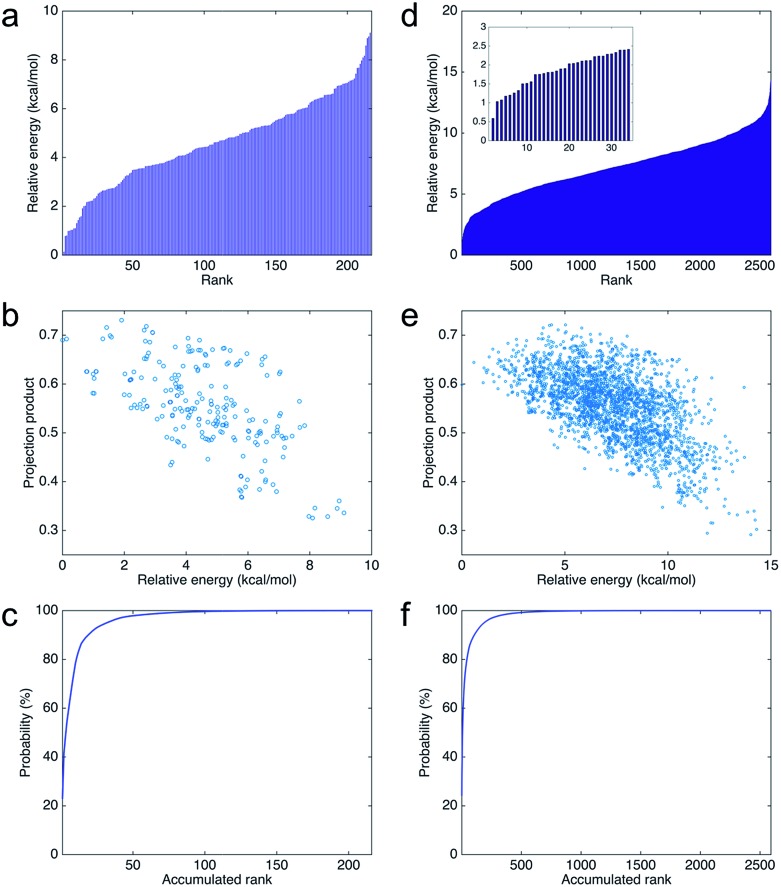
Statistical characteristics of data for trimers and tetramers. (a) The relative energies and ranks of the 216 trimers, depending on their relative energies, from the most to least stable one. (b) The projection products against the relative energies of all trimers. (c) The cumulative Boltzmann distribution function for all trimers. (d) The relative energies and ranks of the 2592 tetramers, depending on their relative energies, from the most to least stable one. The inner figure zooms in the relative energies of the top 34 ranked tetramers. (e) The projection products against the relative energies of all tetramers. (f) The cumulative Boltzmann distribution function for all tetramers.

Fig. 8a shows the relative energies and ranks of the 216 trimers, from the most to least stable one. Note that the relative energy increases dramatically as the rank goes up. Fig. 8b plots the projection products against the relative energies of all trimers. The projection products quantify the planarity of DHI oligomers, ranging from zero to one, being the later a completely planar molecular structure (see ESI†). The distribution of points in the cloud shows a trend that the projection product decreases as the relative energy increases. The result implies that those trimers with more planar molecular structures present lower relative energies, and thus they are more stable. Fig. 8c shows the probability of finding certain trimers in a molecular mixture of all trimers based on the Boltzmann distribution. The top 12 stable trimers shown in Fig. 6 represent 97% of the population of all trimers. The curve is calculated using the Boltzmann distribution function as:1
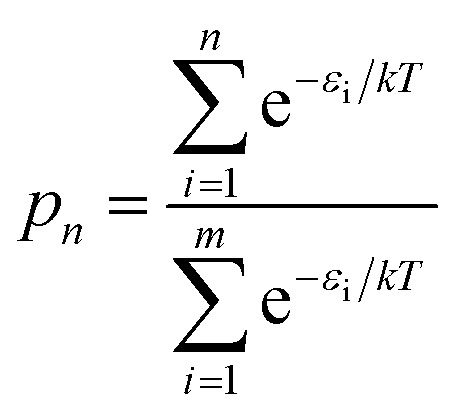
where *p*_*n*_ is the probability of finding the molecular structures that are made of the top *n* (*e.g.*, from 1 to 216 for the trimers) stable molecules including their higher energy conformations, *m* is the total number of molecules (*e.g.*, 216 for the trimers), *ε*_i_ is the energy of molecule-i (here we use its relative energy), *k* is the Boltzmann constant, and *T* is the temperature (here we use 300 K). Fig. 8d shows the relative energies and ranks of the 2592 tetramers. Note that the relative energy increases even more dramatically in the beginning compared to that in the case of trimers (Fig. 8a). The inner figure in Fig. 8d zooms in the relative energies of the top 34 tetramers. These 34 tetramers include the top 16 stable tetramers shown in Fig. 7 and their higher energy conformations. Note that these tetramers are significantly more stable (with lower energy) than the other tetramers. Therefore, even though there are 162 structural unique tetramers, the majority species of tetramers in PDA and eumelanin could be only a few. A possible explanation is that as oligomers become larger there are more constraints for adding a new monomer. For example, when two monomers are trying to form a dimer, these two monomers can freely rotate against each other, and thus they can adjust their relative positions to the lowest energy configuration. On the other hand, when a new monomer is trying to form a tetramer with a trimer, the monomer is not only interacting with the terminal monomer to which it is attaching but also interacting with the other two monomers *via* non-covalent interactions such as the van der Waals and charge interactions. As a result, the new monomer has to compromise on a higher energy configuration when it attaches to an oligomer. In other words, the degree of freedom for adding a new monomer to an oligomer decreases as the number of DHI units in the oligomer increases due to overlapping electron clouds (steric effects). This result explains the existence of relatively small degrees of polymerization in PDA and eumelanin.

Fig. 8e shows the projection products and relative energies of all tetramers. In the figure, there is a clear trend showing that more planar tetramers present lower relative energies, and thus they are more likely to exist. This finding agrees with the fact that PDA and eumelanin are composed of nearly planar oligomers. Note that the planarity in the molecular structures of PDA and eumelanin is essential to explain the physiochemical properties of eumelanin-like materials. Only if the oligomers are nearly planar, layered aggregates made of stacked oligomers *via* π–π interactions can be observed in experiments.^24,46^ Furthermore, only if the oligomers are able to stack together closely to form so-called secondary structures, the excitonic effects among the oligomers can be strong enough to produce the broadband absorption spectrum of PDA and eumelanin.^47^ Fig. 8f shows the probability of finding certain tetramers in a molecular mixture of all tetramers. Based on the Boltzmann distribution, the top 16 stable tetramers show in Fig. 7 represent 77% of the population of all tetramers. This result suggests that the majority species of tetramers is quite repetitive even though the number of probable tetramers is large.

### Self-assembly mechanism

**Fig. 9:**
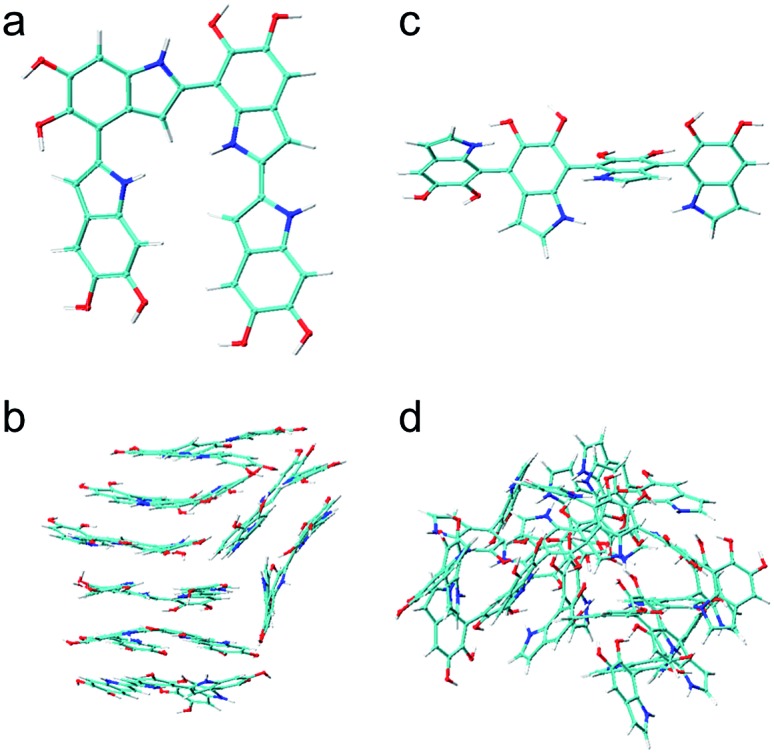
Molecular and aggregated structures of the most and least stable tetramers. (a) The molecular structure of the most stable tetramer. (b) The layered aggregate formed by 8 of the most stable tetramers shown in (a). (c) The molecular structure of the least stable tetramer. (d) The amorphous aggregate formed by 8 of the least tetramers shown in (c).

Using nearly planar molecular models to simulate the self-assembly mechanism of PDA and eumelanin is important. Fig. 9a shows the DFT optimized geometry of the most planar tetramer (Fig. 7o) among the top 16 stable tetramers (Fig. 7). This tetramer not only is one of the most stable tetramers identified in this work but also has a very high projection product of 0.68 (Fig. 8e). While eight of the tetramers are placed in a simulation box, they quickly stack together to form a layered aggregate shown in Fig. 9b in the simulation (see Computational details section). This type of layered aggregates is one of the most important structural features of eumelanin-like materials. Note that only nearly planar molecular models are able to form such aggregate structures. For example, Fig. 9c shows the DFT optimized geometry of the tetramer that has the lowest projection product of 0.29 (Fig. 8e). Interestingly, this tetramer is also one of the least stable tetramers, which ranks 2589 of 2592. With the same simulation setup, as eight of the tetramers are placed in a simulation box, they also quickly aggregate in the simulation. However, they do not stack together to form a layered aggregate, instead, they form an amorphous structure shown in Fig. 9d. This result clearly demonstrates that choosing more realistic molecular models of PDA and eumelanin for simulations is crucial to get meaningful results.

## Conclusions

In this work, we discuss possible reaction mechanisms of DHI and identify its most reactive positions. For the first time, a first-principle approach is applied to provide a quantum chemical explanation for the existence of certain preferential connections between DHI monomers. Using reactivity descriptors such as the Fukui function and spin densities calculations, we support some of the assumed mechanisms of the DHI polymerization. These results are then adopted as an input for the brute-force algorithmic generator that generates all probable chemical isomers for small DHI oligomers. From a total number of nearly 3000 isomers, the most stable dimers, trimers, and tetramers of DHI are selected to propose molecular models that will help more accurate modeling of PDA and eumelanin. The relative energies between molecular models reported in this work can be applied to estimate a proper ratio of these molecules in simulations. We also provide fundamental explanations for some important structural features of PDA and eumelanin, such as the structural planarity and small size of oligomers. It is important to remark that we have evaluated DHI oligomers only up to tetramers and have not considered different redox forms due to computational limitations. Note that PDA and eumelanin contain a portion of oligomers larger than tetramers, and oxidation has a profound impact on the molecular structures. Consequently, this work cannot completely elucidate the controversial molecular structures of PDA and eumelanin. However, this work provides some new possible findings and explanations to the structural properties of eumelanin-like materials. Most importantly, the theoretical framework and brute-force algorithmic generator in this work can be easily applied to more realistic situations such as to explore larger oligomers (*e.g.*, pentamers and hexamers), to consider different redox forms (*e.g.*, NQ, MQ, and IQ), and to include DHICA, in future studies when more computational resources are available.

## Computational details

### Reactivity and spin density

The reactivity of various DHI monomers and oligomers has been characterized using the Fukui function dual descriptor within the framework of conceptual DFT.^48^ The Fukui function describes how the electron density changes in response to an increase or a decrease in the number of electrons. Accordingly, it is employed to predict the preferred site for either a nucleophilic or electrophilic attacks. The population of unpaired electrons for different sites in the radicals has been quantified with electron spin densities, for which unrestricted open shell DFT calculations are performed.

### Brute-force algorithmic generator

**Fig. 10:**
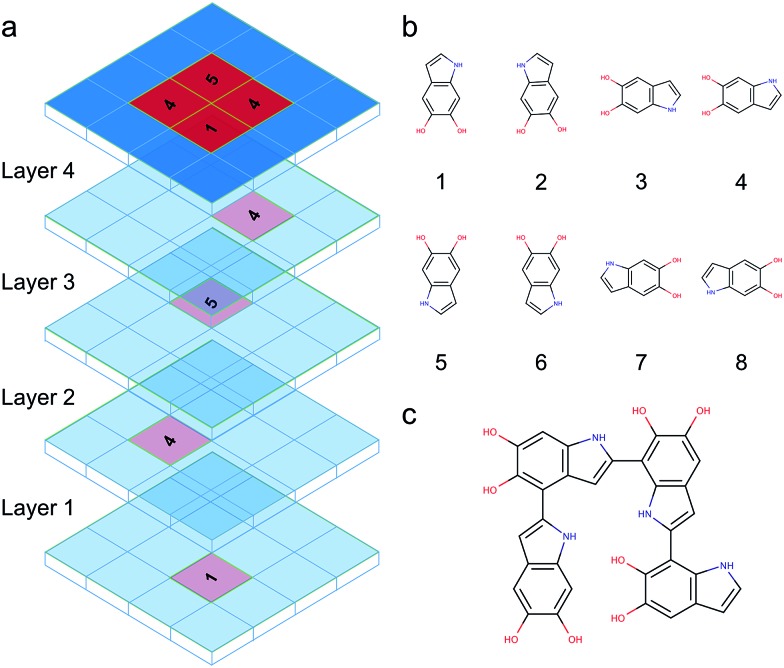
Brute-force algorithmic generator using multi-layers of checkerboard plates to generate different DHI oligomers. (a) Four layers of checkerboard plates for generating a DHI tetramer. The red blocks represent the elements that are occupied for generating a DHI tetramer and the blue blocks represent available elements. The numbers on the red blocks represent the orientation of DHI monomers shown in (b). Eight different orientations of a DHI monomer are considered in the algorithm. (c) The molecular structure of a tetramer that is generated according to the four layers of checkerboard plates shown in (a).

DHI oligomers are composed of various numbers of DHI units with different configurations. In this work, we use multi-layers of checkerboard plates to represent different polymerization degrees of DHI oligomers. For example, a tetramer can be generated with four layers of checkerboard plates (Fig. 10a). We consider eight different orientations of a DHI monomer (Fig. 10b) to generate DHI oligomers. Orientation 1 is the default orientation. Orientation 2 is a flipped structure of orientation 1. Orientation 3 is a 90 degree clockwise-rotated structure of orientation 1. Orientation 4 is a flipped structure of orientation 3. Similarly, orientation 5 is a 180 degree clockwise-rotated structure of orientation 1. Orientation 6 is a flipped structure of orientation 5. Orientation 7 is a 270 degree clockwise-rotated structure of orientation 1. Orientation 8 is a flipped structure of orientation 7. With these eight different orientations, we can generate all probable DHI oligomers by assembling two or more DHI monomers with different orientations on checkerboard plates. Take dimers for an example, the first monomer in the brute-force algorithmic generator is the default orientation, orientation 1, which is placed on the first layer of a checkerboard plate. Note that it makes no difference which orientation of a DHI monomer is chosen as the first monomer. The brute-force algorithmic generator can generate all probable DHI oligomers no matter which orientation of a DHI monomer is chosen as the starting point. According to our results, the most reactive positions of DHI are the 2-, 4-, and 7-position, and thus we assume that there are three reactive positions in DHI. The first reactive position considered in the brute-force algorithmic generator is the 7-position. There are six orientations of a DHI monomer (*i.e.*, orientation 1, 2, 5, 6, 7, and 8) can form a covalent bond with orientation 1 at the 7-position. The second reactive position considered is the 2-position. There are also six orientations of a DHI monomer (*i.e.*, orientation 3, 4, 5, 6, 7, and 8) can form a covalent bond with orientation 1 at the 2-position. The third reaction position considered is the 4-position. Similarly, there are also six orientations of a DHI monomer (*i.e.*, orientation 1, 2, 3, 4, 5, and 6) can form a covalent bond with orientation 1 at the 4-position. Consequently, a total of 18 combinations of positions and orientations can be generated on the second layer of checkerboard plates. Combining the first and second layers of checkerboard plates, a total of 18 dimers can be generated. Fig. S3† shows the checkerboard representations of these 18 dimers. The numbers on the checkerboard plates indicate the orientations of DHI monomers, where the corresponding molecular structures are shown in Fig. S1.† While generating trimers, the third layer of checkerboard plates is added to connect the third DHI monomer with the second DHI monomer. Since the second DHI monomer is already formed a covalent bond with the first DHI monomer, there are only two reactive positions available to form a covalent bond with the third DHI monomer. As a result, a total of 12 combinations of positions and orientations can be generated on the third layer of checkerboard plates for an individual dimer. Combining the first, second, and third layers of checkerboard plates, a total of 216 trimers can be generated. The molecular structures of these 216 trimers are shown in Fig. S2.† Similarly, a total of 2592 tetramers can be generated in the brute-force algorithmic generator. Fig. 10c shows the molecular structure of a tetramer generated according to the four layers of checkerboard plates shown in Fig. 10a. Finding the lowest energy conformation of an oligomer is critical for comparing its energy with other oligomers. Note that the brute-force algorithmic generator not only can generate all probable structural unique oligomers but also can generate all different conformations, which oligomers can adopt by rotating about covalent (sigma) bonds. As a result, the conformational analysis is done at the same time while we evaluate the energies of different conformations of oligomers.

### Atomistic modeling and equilibration

Full atomistic MD simulations are performed to relax and equilibrate the initial molecular structures of DHI oligomers generated using the brute-force algorithmic generator. The large-scale atomic/molecular massively parallel simulator (LAMMPS)^49^ with the consistent valence force field (CVFF) are adopted in this work. The CVFF has been widely applied in modeling organic molecules with aromatic rings such as benzene, polydopamine,^46,47,50^ and caffeine co-crystals.^51^ Energy minimization using the conjugate gradient (CG) algorithm is performed to relax the initial molecular structures. After energy minimization, MD simulations are performed to further equilibrate the molecular structures. The integration time step is 1.0 fs and the nonbonding interactions (12–6 Lennard-Jones and coulombic interactions) are computed with a cutoff of 12 Å. The MD equilibration includes 10 iterations of equilibrations in order to find the most stable geometry of each molecule. Each iteration contains four steps of the NVT ensembles. In the first step, a molecule is equilibrated with the NVT ensemble at a temperature starting from 1.0 K and increasing linearly to 300 K in 1.0 ps. In the second step, the molecule is equilibrated with the NVT ensemble at 300 K for 1.0 ps. In the third step, the molecule is equilibrated with the NVT ensemble at a temperature starting from 300 K and decreasing linearly to 1.0 K in 1.0 ps. In the final step, the molecule is equilibrated with the NVT ensemble at 1.0 K for 1.0 ps. A Langevin thermostat is performed to control the temperature. When the fourth step of the NVT ensembles is finished, the molecule is then relaxed with energy minimization again to calculate its energy. After that, its geometry and the corresponding energy are recorded. This set of the NVT ensembles is repeated 10 times. As a result, a total of 10 geometries and the corresponding energies are recorded for each molecule. The geometry with the lowest energy is then selected for further geometry optimizations with DFT. The MD equilibration scheme is shown in Fig. S4.†

### Geometry optimization

The geometries obtained from the MD equilibration are further optimized with DFT using ORCA quantum chemistry package.^52^ The dispersion correction DFT-D3 with Becke–Johnson damping (D3BJ)^53^ is implemented. In addition, PDA and eumelanin are both synthesized in water solution. Therefore, the SMD model,^54^ a continuum solvation model based on the quantum mechanical charge density of a solute molecule interacting with a continuum description of the solvent, is adopted to take water effects into account. The B3LYP^36,37^ functional is adopted for optimizing the molecular structures of the dimers, together with the def2-QZVP^38^ basis set. However, the B3LYP/def2-QZVP is too expensive for optimizing the molecular structures of the trimers and tetramers. As a result, the BLYP^43,44^ functional together with the def2-SVP^38^ basis set is adopted for optimizing the trimers and tetramers. A benchmark study is conducted to evaluate the accuracy of using the BLYP/def2-SVP for optimizing the molecular structures of DHI oligomers. The 18 dimers (Fig. S1†) are used for the benchmark. Fig. S5† shows the relative energies of the 18 dimers compared to the dimer-9 (with lowest energy) using the B3LYP/def2-QZVP and BLYP/def2-SVP. The reference values are calculated using the B3LYP/def2-QZVP. The large def2-QZVP basis set minimizes the basis set error and provides the results (blue bars) near the basis set limit for the B3LYP. On the other hand, the BLYP/def2-SVP yields very different results (yellow bars) compared to the reference values. The difference mainly comes from the so-called basis set superposition error (BSSE) when applying small basis sets.^55^ In this work, we adopt the recently developed geometrical counterpoise correction (gCP)^56^ to circumvent the BSSE. The improvement of using the BSSE-correction gCP is significant. The results (green bars) calculated using the BLYP/def2-SVP/gCP are quite close to the reference values (blue bars). The energy differences between using the B3LYP/def2-QZVP and BLYP/def2-SVP/gCP are within 1 kcal mol^–1^. Since the BLYP/def2-SVP/gCP can provide acceptable results and it is at least 500 times faster than the more accurate B3LYP/def2-QZVP, the BLYP/def2-SVP/gCP is adopted for the geometry optimizations of the trimers and tetramers. The energies obtained in the DFT calculations are used to benchmark the molecular structures of DHI oligomers.

### Self-assembly modeling

Two tetrameric models are used to study the self-assembly mechanism. For each model, eight tetramers are separated by a distance larger than 25 Å in the initial configuration, to ensure that there are no intermolecular interactions. After energy minimization, the system is equilibrated with the NVT ensemble at 300 K for 1.0 ns. The snapshots in Fig. 9b and d are captured after the simulations are finished.

## References

[cit1] Lee H., Dellatore S. M., Miller W. M., Messersmith P. B. (2007). Science.

[cit2] Zhong C., Gurry T., Cheng A. A., Downey J., Deng Z., Stultz C. M., Lu T. K. (2014). Nat. Nanotechnol..

[cit3] Qin Z., Buehler M. J. (2013). Nat. Commun..

[cit4] Qin Z., Buehler M. J. (2014). J. Mech. Phys. Solids.

[cit5] Qin Z., Buehler M. (2012). Appl. Phys. Lett..

[cit6] Liu Y., Ai K., Lu L. (2014). Chem. Rev..

[cit7] d'Ischia M., Napolitano A., Ball V., Chen C. T., Buehler M. J. (2014). Acc. Chem. Res..

[cit8] Sheng W., Li B., Wang X., Dai B., Yu B., Jia X., Zhou F. (2015). Chem. Sci..

[cit9] Ball V., Del Frari D., Michel M., Buehler M. J., Toniazzo V., Singh M. K., Gracio J., Ruch D. (2012). BioNanoScience.

[cit10] Ku S. H., Lee J. S., Park C. B. (2010). Langmuir.

[cit11] Gao H., Sun Y., Zhou J., Xu R., Duan H. (2013). ACS Appl. Mater. Interfaces.

[cit12] Li Z., Zhang X., Wang S., Yang Y., Qin B., Wang K., Xie T., Wei Y., Ji Y. (2016). Chem. Sci..

[cit13] Mu J., Hou C., Wang H., Li Y., Zhang Q., Zhu M. (2015). Sci. Adv..

[cit14] Lynge M. E., van der Westen R., Postma A., Stadler B. (2011). Nanoscale.

[cit15] Qiang W., Li W., Li X., Chen X., Xu D. (2014). Chem. Sci..

[cit16] Wang B., Wang G., Zhao B., Chen J., Zhang X., Tang R. (2014). Chem. Sci..

[cit17] Kang S. M., Park S., Kim D., Park S. Y., Ruoff R. S., Lee H. (2011). Adv. Funct. Mater..

[cit18] Cheng J., Moss S. C., Eisner M., Zschack P. (1994). Pigm. Cell Res..

[cit19] Cheng J., Moss S. C., Eisner M. (1994). Pigm. Cell Res..

[cit20] Díaz P., Gimeno Y., Carro P., González S., Schilardi P. L., Benítez G., Salvarezza R. C., Creus A. H. (2005). Langmuir.

[cit21] Zajac G., Gallas J., Cheng J., Eisner M., Moss S., Alvarado-Swaisgood A. (1994). Biochim. Biophys. Acta, Gen. Subj..

[cit22] Zajac G. W., Gallas J. M., Alvarado-Swaisgood A. E. (1994). J. Vac. Sci. Technol., B: Microelectron. Nanometer Struct.--Process., Meas., Phenom..

[cit23] Clancy C. M., Nofsinger J. B., Hanks R. K., Simon J. D. (2000). J. Phys. Chem. B.

[cit24] Watt A. A., Bothma J. P., Meredith P. (2009). Soft Matter.

[cit25] Kaxiras E., Tsolakidis A., Zonios G., Meng S. (2006). Phys. Rev. Lett..

[cit26] Meng S., Kaxiras E. (2008). Biophys. J..

[cit27] Della Vecchia N. F., Avolio R., Alfè M., Errico M. E., Napolitano A., d'Ischia M. (2013). Adv. Funct. Mater..

[cit28] d'Ischia M., Wakamatsu K., Napolitano A., Briganti S., Garcia-Borron J. C., Kovacs D., Meredith P., Pezzella A., Picardo M., Sarna T. (2013). Pigm. Cell Melanoma Res..

[cit29] Dreyer D. R., Miller D. J., Freeman B. D., Paul D. R., Bielawski C. W. (2012). Langmuir.

[cit30] Tran M. L., Powell B. J., Meredith P. (2006). Biophys. J..

[cit31] Hong S., Na Y. S., Choi S., Song I. T., Kim W. Y., Lee H. (2012). Adv. Funct. Mater..

[cit32] Okuda H., Yoshino K., Wakamatsu K., Ito S., Sota T. (2014). Pigm. Cell Melanoma Res..

[cit33] Yang J., Cohen Stuart M. A., Kamperman M. (2014). Chem. Soc. Rev..

[cit34] Land E. J., Ramsden C. A., Riley P. A. (2003). Acc. Chem. Res..

[cit35] Kalyanaraman B., Felix C., Sealy R. (1985). Environ. Health Perspect..

[cit36] Becke A. D. (1993). J. Chem. Phys..

[cit37] Becke A. D. (1993). J. Chem. Phys..

[cit38] Weigend F., Ahlrichs R. (2005). Phys. Chem. Chem. Phys..

[cit39] Panzella L., Pezzella A., Napolitano A., d'Ischia M. (2007). Org. Lett..

[cit40] d'Ischia M., Napolitano A., Pezzella A., Land E. J., Ramsden C. A. and Riley P. A., in Advances in Heterocyclic Chemistry, ed. R. K. Alan, Academic Press, 2005, vol. 89, pp. 1–63.

[cit41] Liebscher J. r., Mrówczyński R., Scheidt H. A., Filip C., Hădade N. D., Turcu R., Bende A., Beck S. (2013). Langmuir.

[cit42] Pezzella A., Napolitano A., d'Ischia M., Prota G. (1996). Tetrahedron.

[cit43] Lee C., Yang W., Parr R. G. (1988). Phys. Rev. B: Condens. Matter Mater. Phys..

[cit44] Becke A. D. (1988). Phys. Rev. A: At., Mol., Opt. Phys..

[cit45] Kim Y. J., Khetan A., Wu W., Chun S. E., Viswanathan V., Whitacre J. F., Bettinger C. J. (2016). Adv. Mater..

[cit46] Chen C. T., Ball V., Gracio J. J. D., Singh M. K., Toniazzo V., Ruch D., Buehler M. J. (2013). ACS Nano.

[cit47] Chen C. T., Chuang C., Cao J. S., Ball V., Ruch D., Buehler M. J. (2014). Nat. Commun..

[cit48] Geerlings P., De Proft F., Langenaeker W. (2003). Chem. Rev..

[cit49] Plimpton S. (1995). J. Comput. Phys..

[cit50] Lin S. C., Chen C. T., Bdikin I., Ball V., Gracio J., Buehler M. J. (2014). Soft Matter.

[cit51] Chen C. T., Ghosh S., Reddy C. M., Buehler M. J. (2014). Phys. Chem. Chem. Phys..

[cit52] Neese F. (2012). Wiley Interdiscip. Rev.: Comput. Mol. Sci..

[cit53] Grimme S., Ehrlich S., Goerigk L. (2011). J. Comput. Chem..

[cit54] Marenich A. V., Cramer C. J., Truhlar D. G. (2009). J. Phys. Chem. B.

[cit55] Kruse H., Goerigk L., Grimme S. (2012). J. Org. Chem..

[cit56] Kruse H., Grimme S. (2012). J. Chem. Phys..

