# Supplementary material for: Polydopamine and eumelanin molecular structures investigated with ab initio calculations
Source: Chem Sci. 2016 Nov 2;8(2):1631–41. doi: 10.1039/c6sc04692d (PMC5364519; doi:10.1039/c6sc04692d)
Supplement: Supplementary file 1 [file SC-008-C6SC04692D-s001.pdf]

Electronic Supplementary Material (ESI) for Chemical Science.
This journal is © The Royal Society of Chemistry 2016

Supplementary Figures

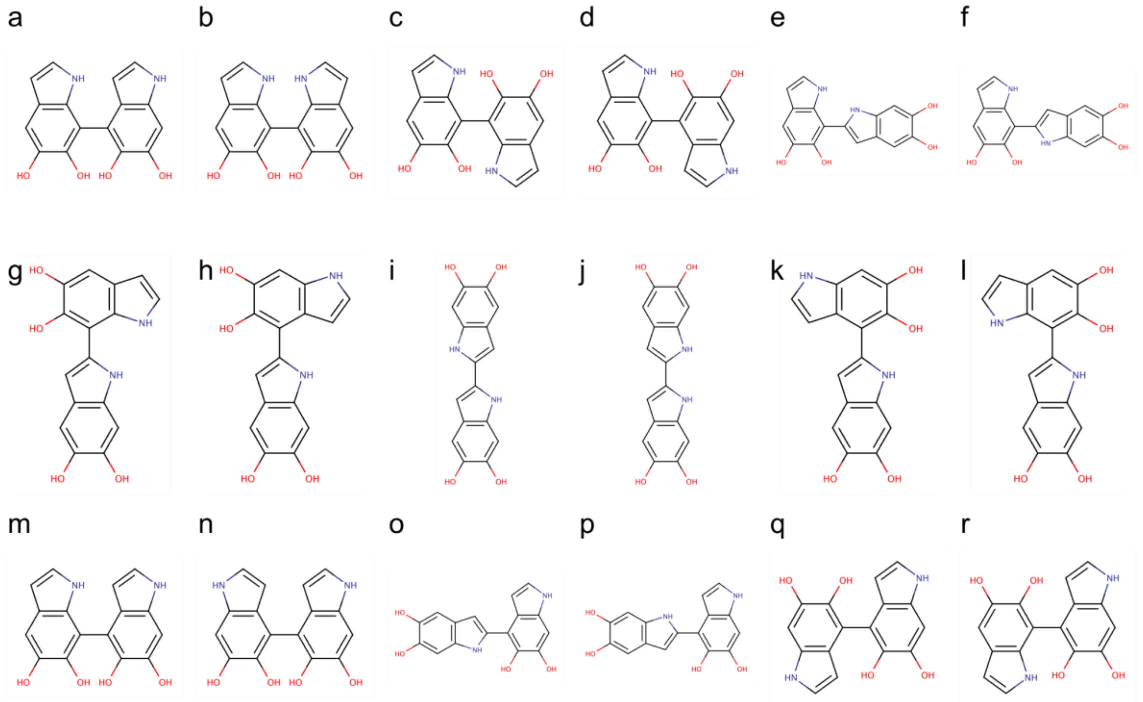

Figure S1 | Molecular structures of dimers. (a) to (r) shows Dimer-1 to Dimer-18, respectively.

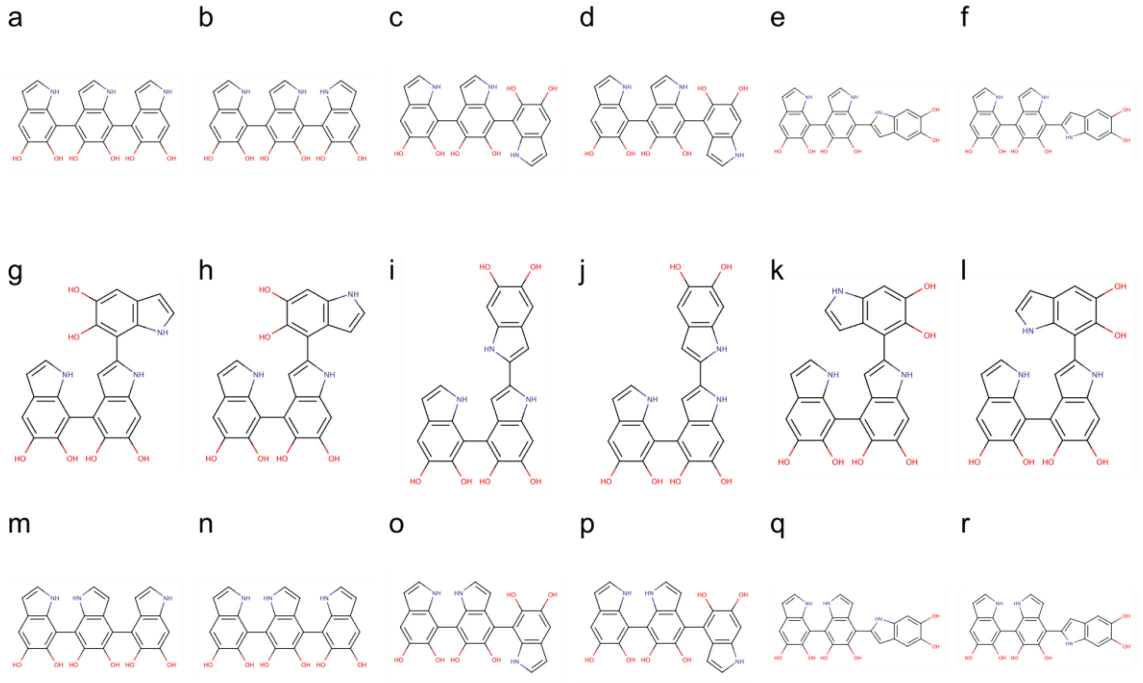

Figure S2-1 | Molecular structures of trimers. (a) to (r) shows Trimer-1 to Trimer-18, respectively.

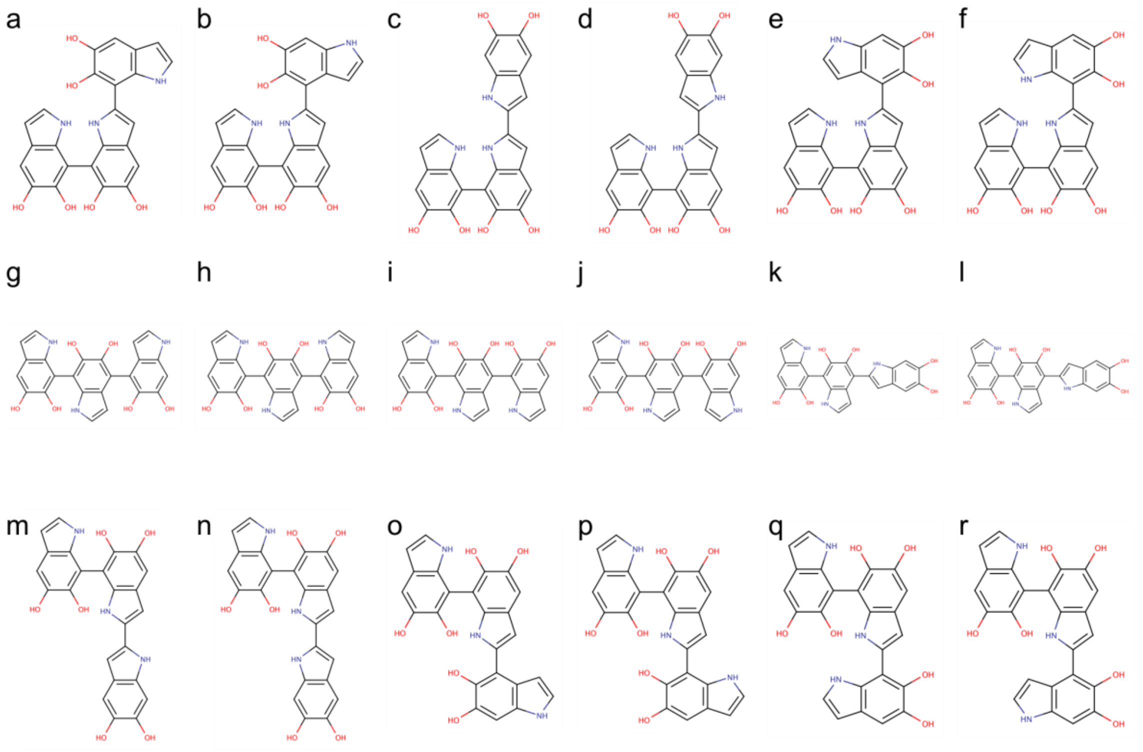

Figure S2-2 | Molecular structures of trimers. (a) to (r) shows Trimer-19 to Trimer-36, respectively.

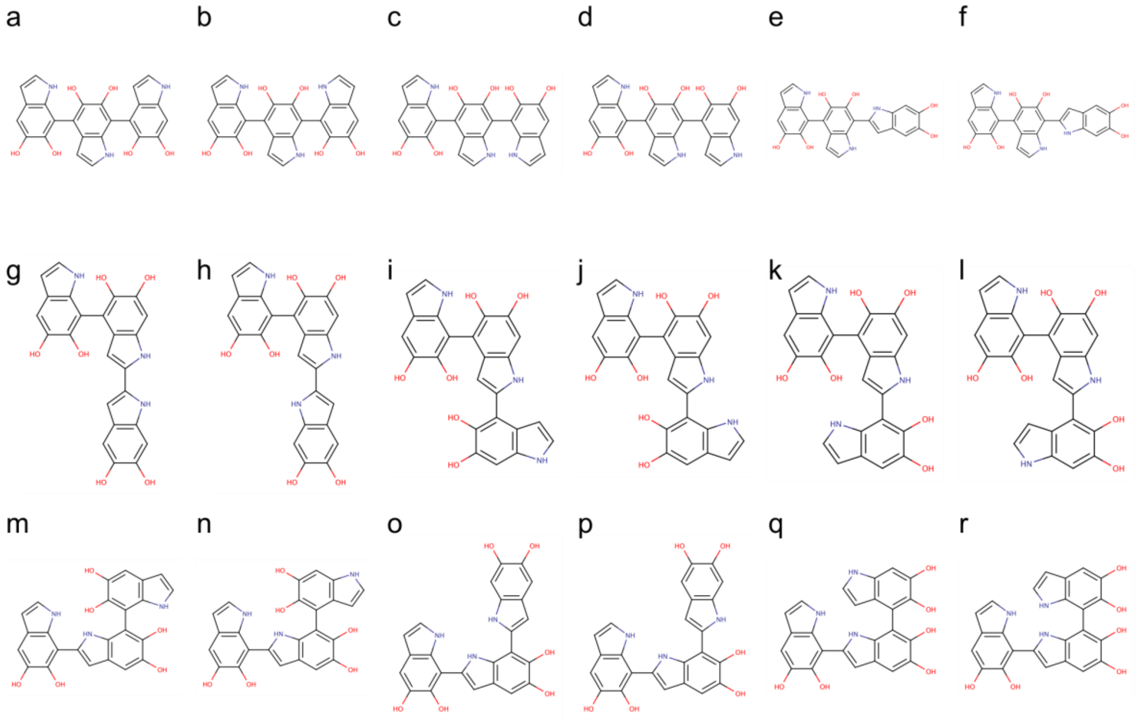

Figure S2-3 | Molecular structures of trimers. (a) to (r) shows Trimer-37 to Trimer-54, respectively.

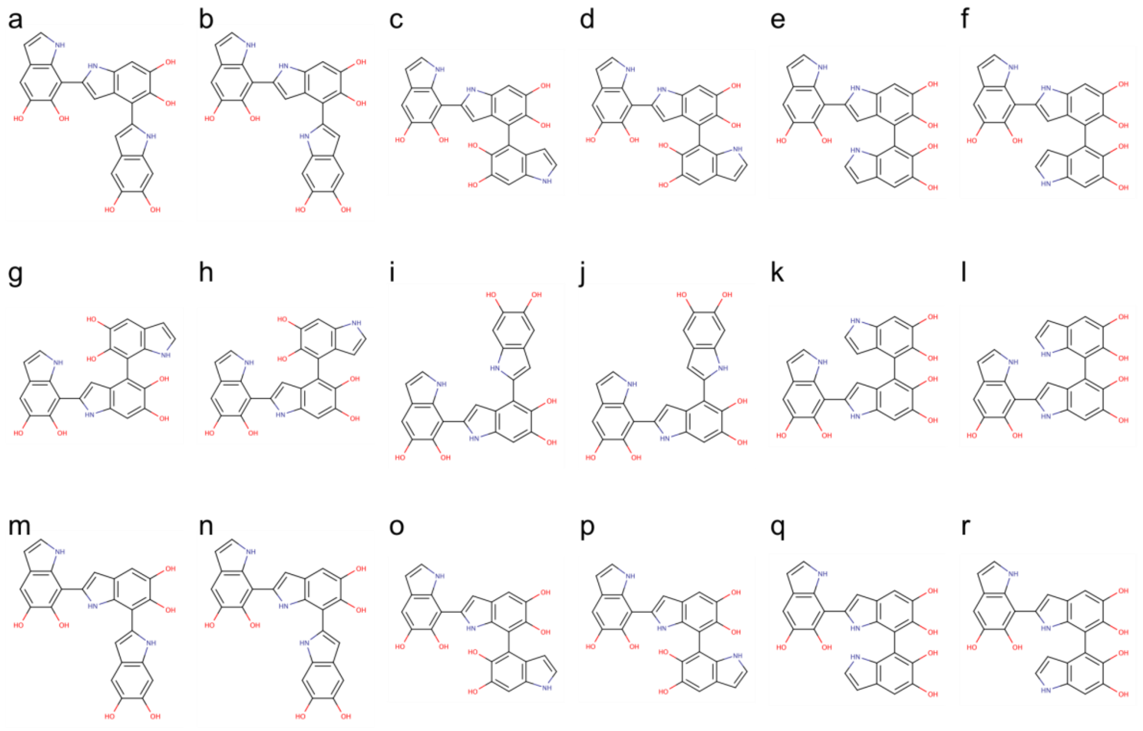

Figure S2-4 | Molecular structures of trimers. (a) to (r) shows Trimer-55 to Trimer-72, respectively.

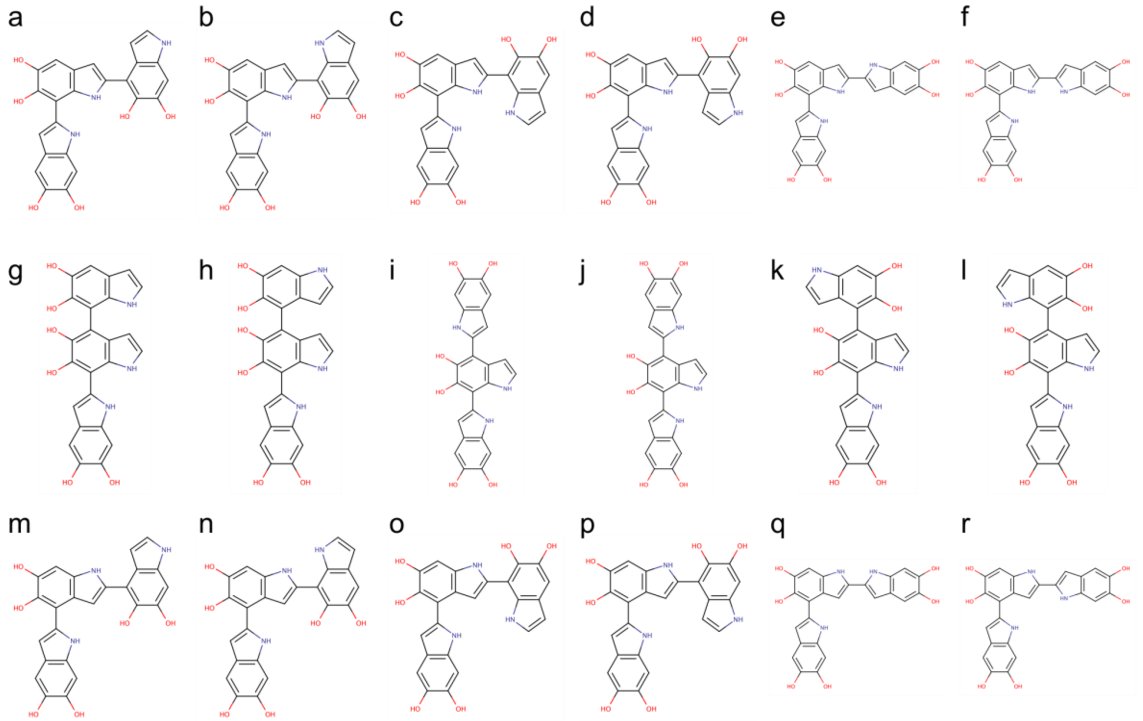

Figure S2-5 | Molecular structures of trimers. (a) to (r) shows Trimer-73 to Trimer-90, respectively.

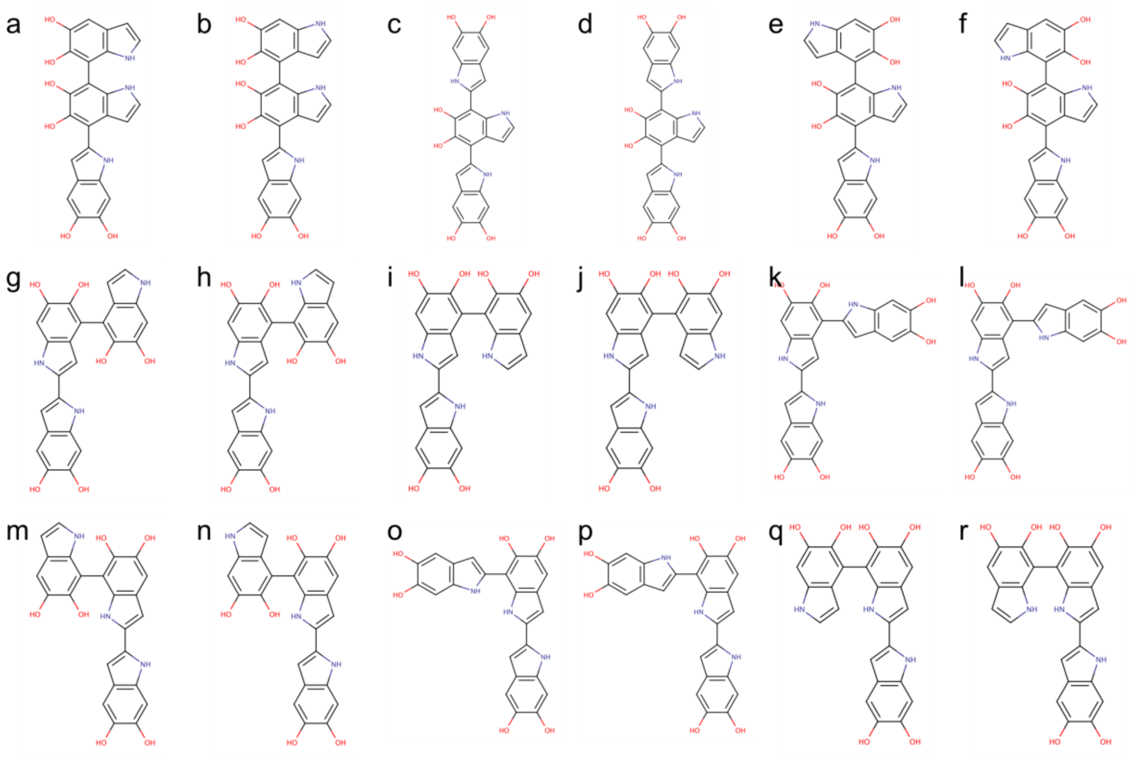

Figure S2-6 | Molecular structures of trimers. (a) to (r) shows Trimer-91 to Trimer-108, respectively.

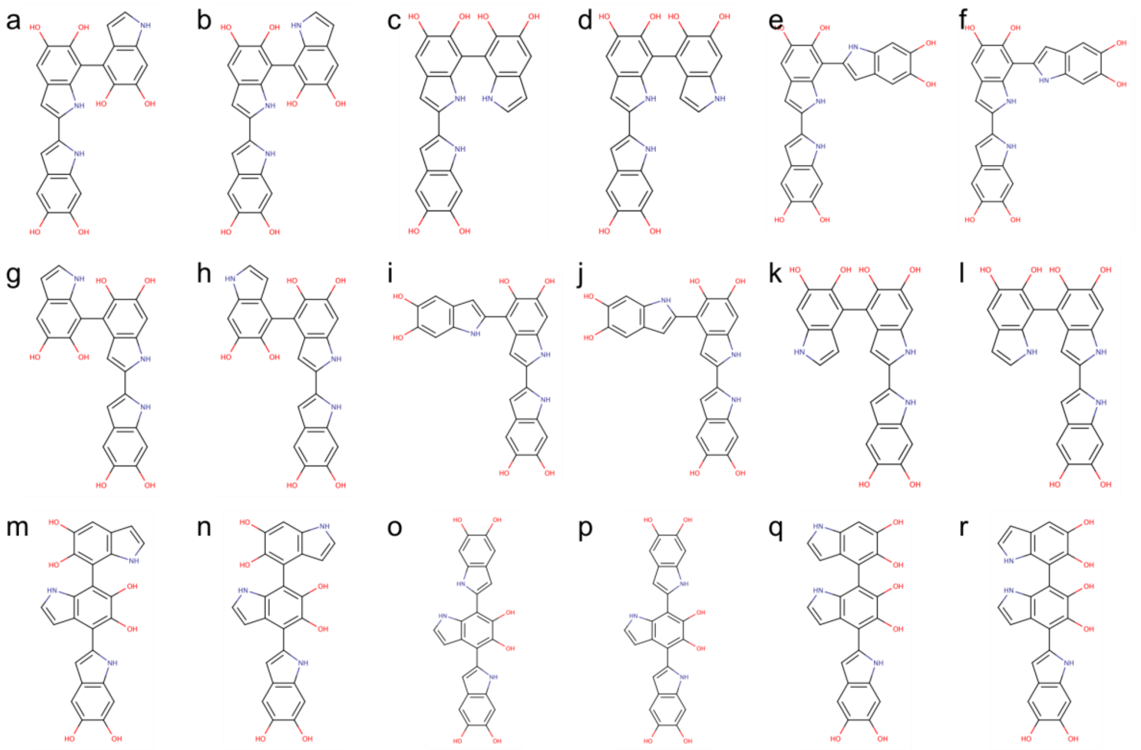

Figure S2-7 | Molecular structures of trimers. (a) to (r) shows Trimer-109 to Trimer-126, respectively.

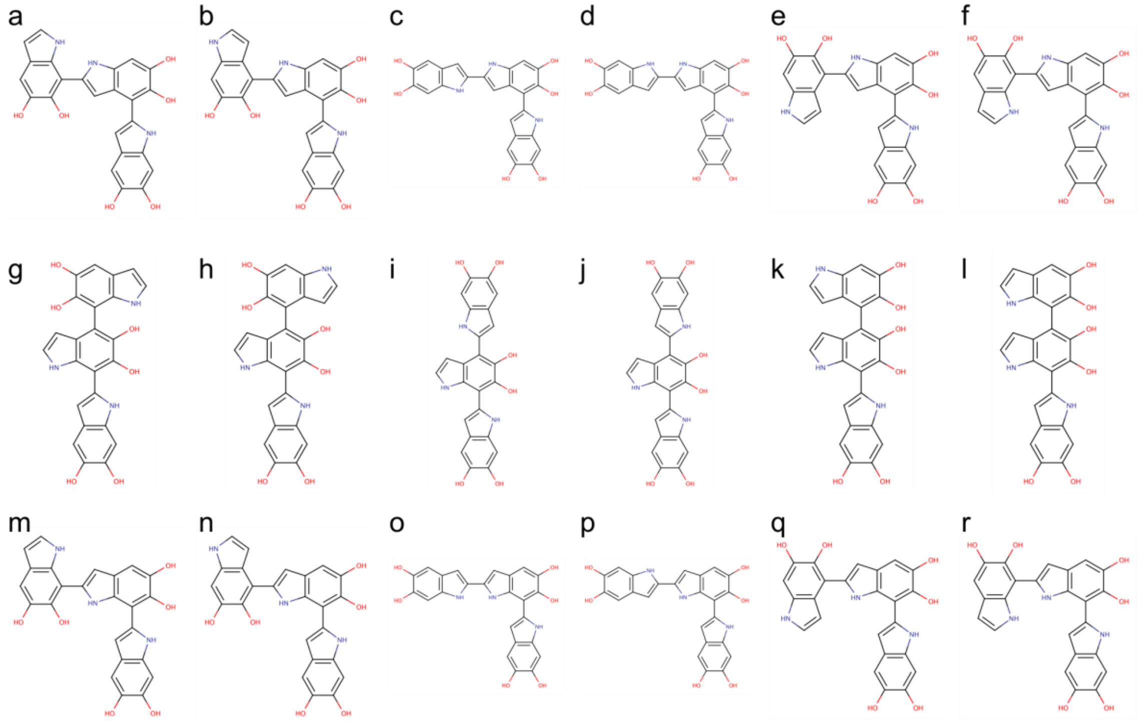

Figure S2-8 | Molecular structures of trimers. (a) to (r) shows Trimer-127 to Trimer-144, respectively.

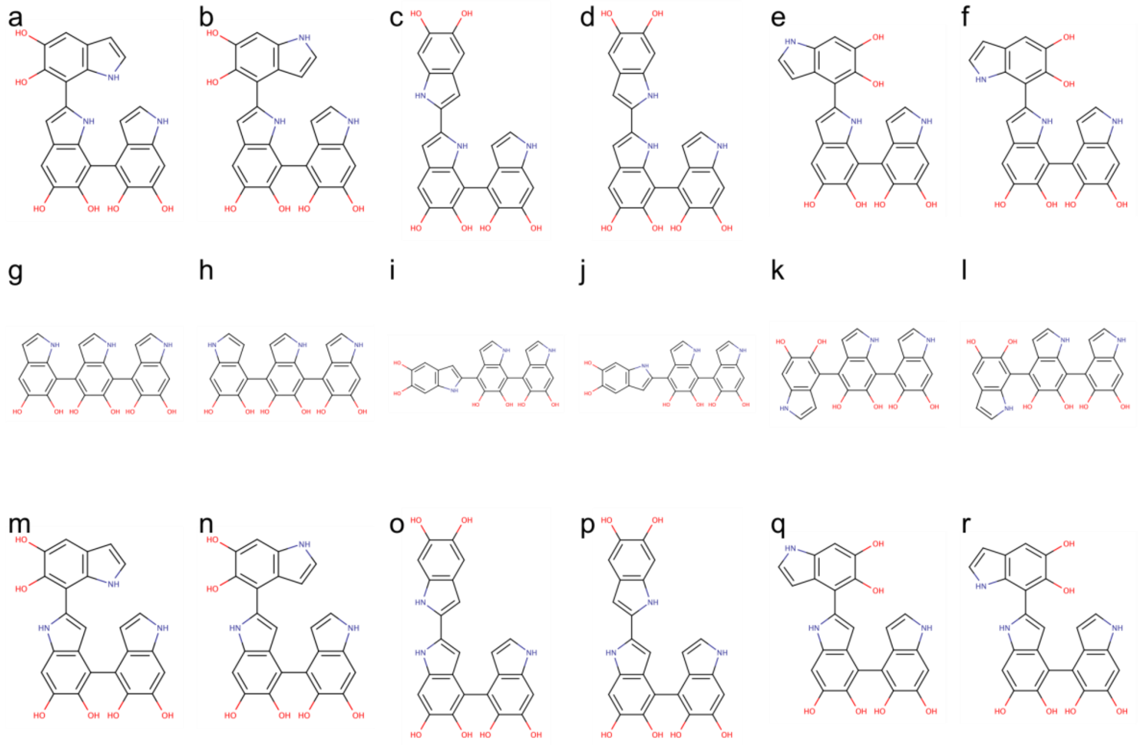

Figure S2-9 | Molecular structures of trimers. (a) to (r) shows Trimer-145 to Trimer-162, respectively.

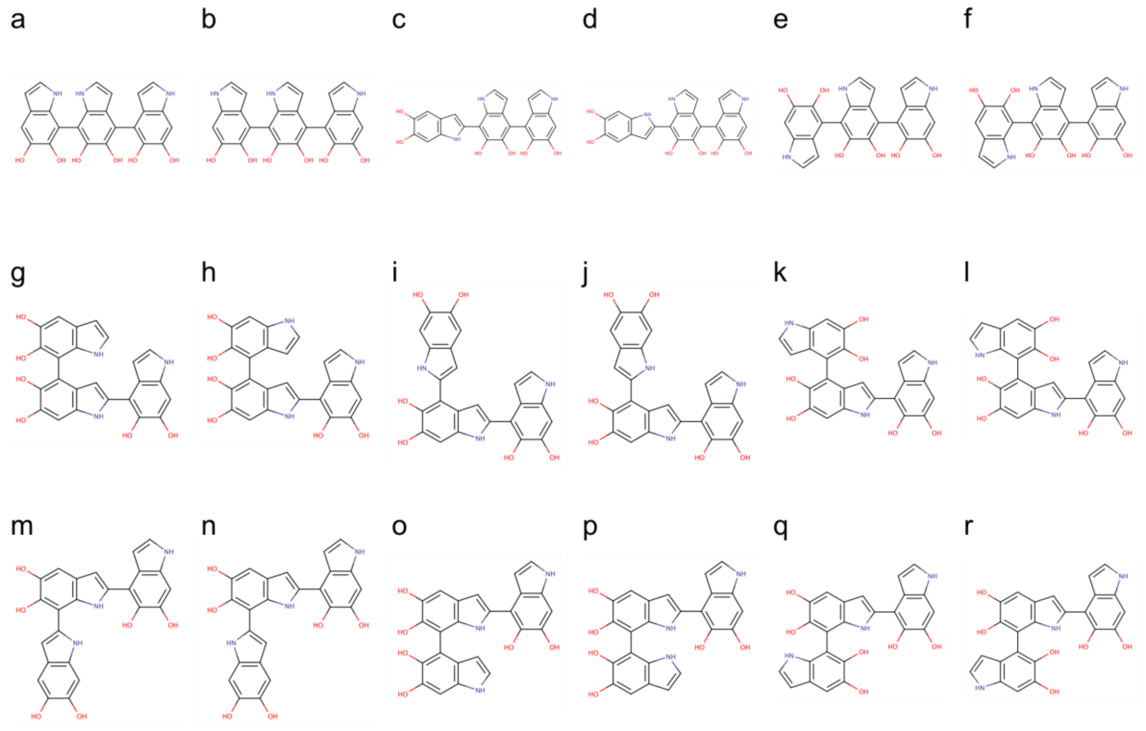

Figure S2-10 | Molecular structures of trimers. (a) to (r) shows Trimer-163 to Trimer-180, respectively.

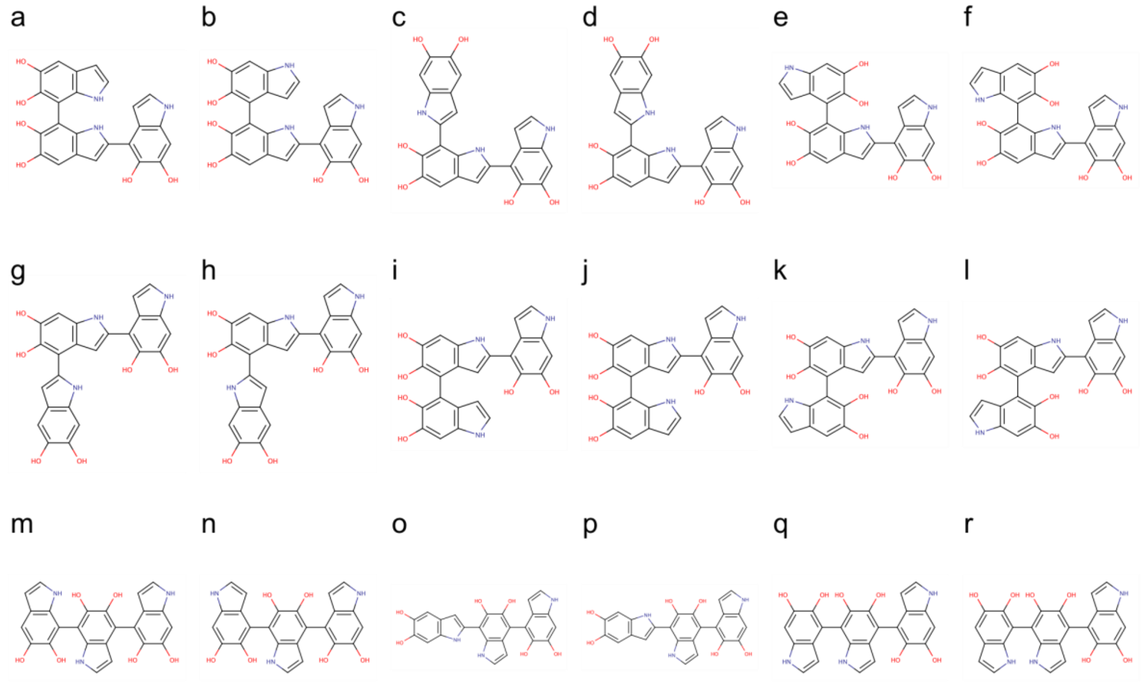

Figure S2-11 | Molecular structures of trimers. (a) to (r) shows Trimer-181 to Trimer-198, respectively.

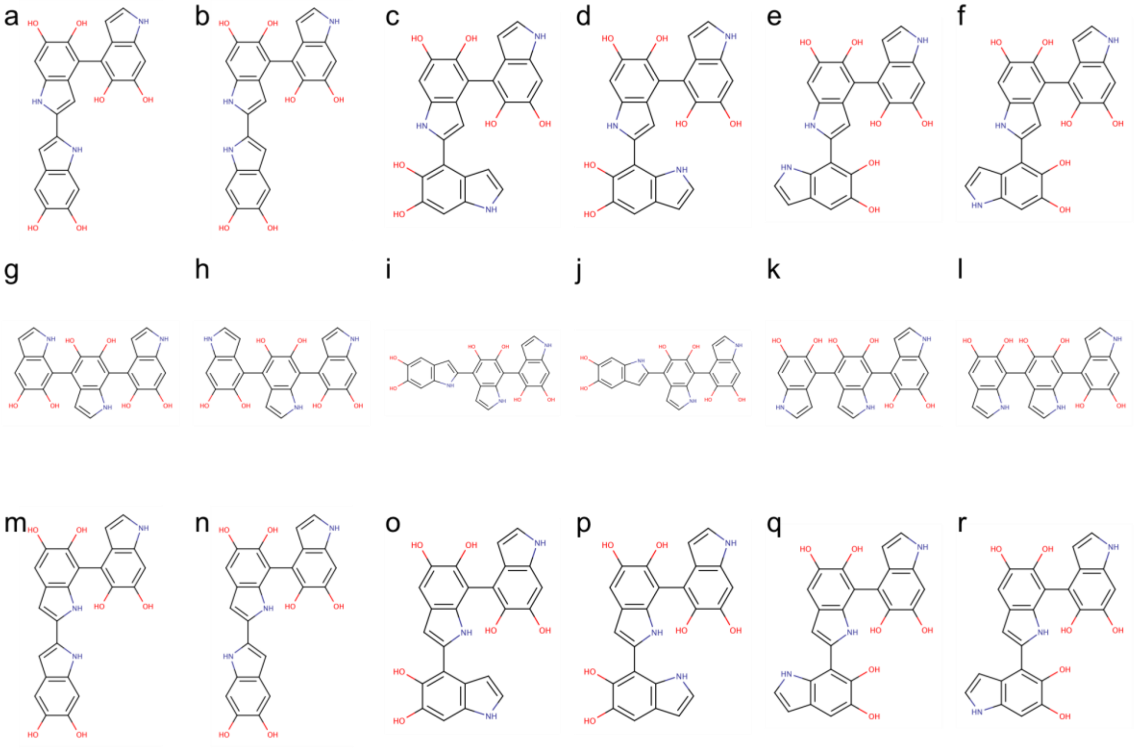

Figure S2-12 | Molecular structures of trimers. (a) to (r) shows Trimer-199 to Trimer-216, respectively.

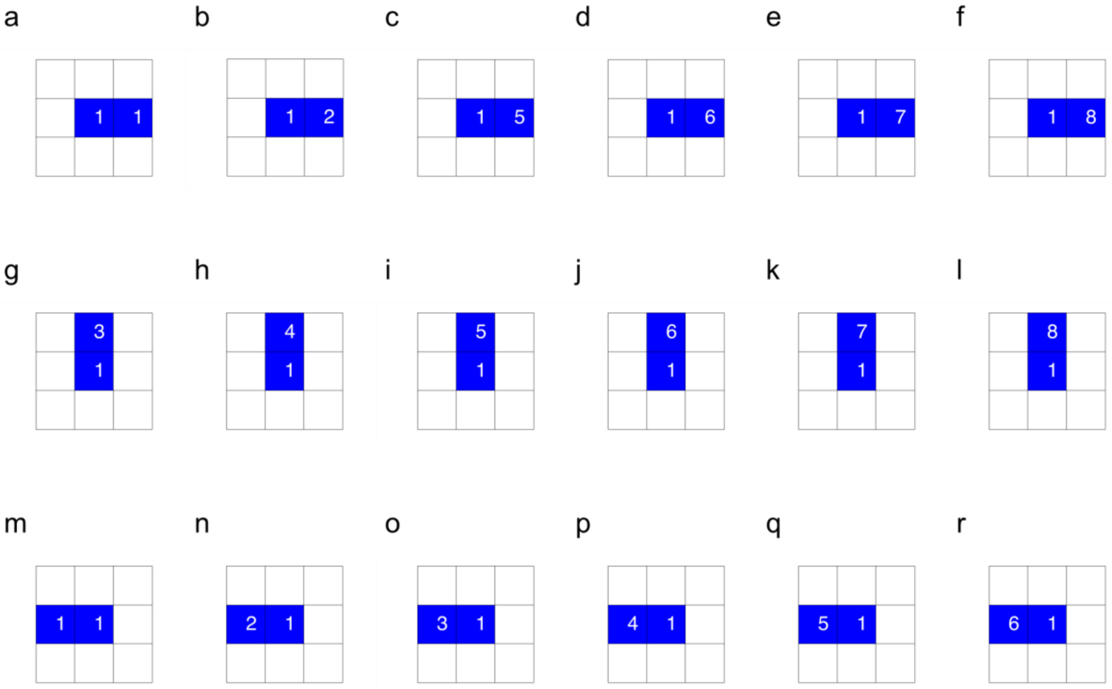

Figure S3 | Checkerboard representations of dimers. (a) to (r) shows Dimer-1 to Dimer-18, respectively.

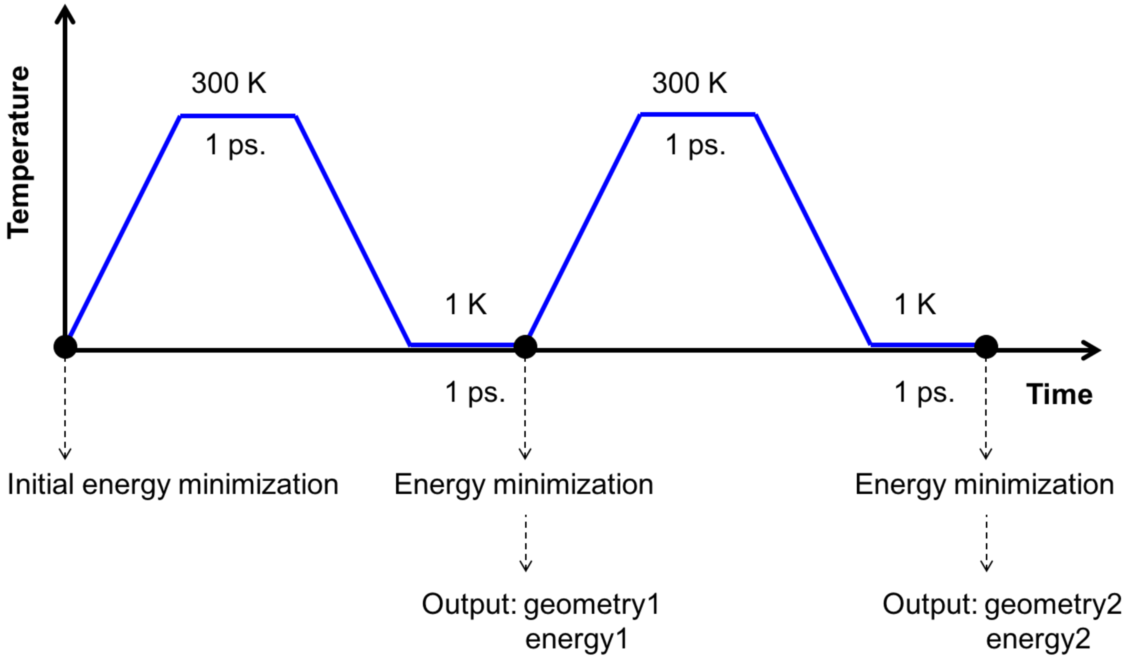

Figure S4 | MD equilibration scheme. The MD equilibration includes 10 iterations. Each iteration generates a geometry and its corresponding energy. The geometry with the lowest energy is adopted for further geometry optimizations with DFT calculations.

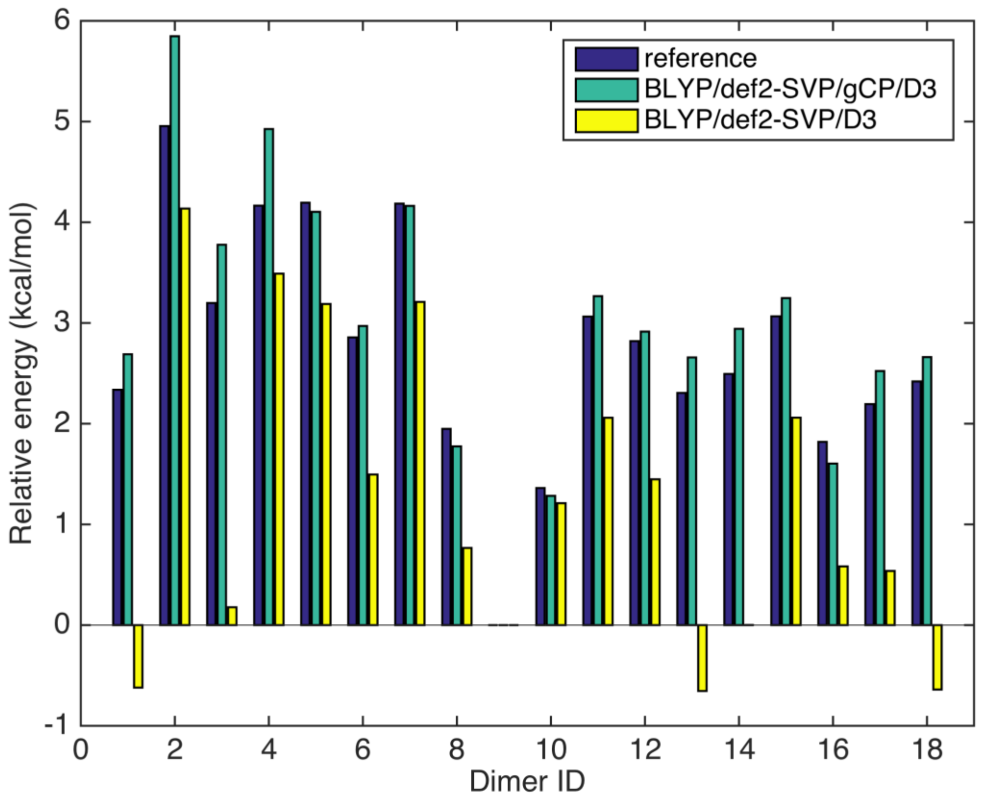

Figure S5 | Relative energies of all dimers obtained from different DFT calculations. The reference values (blue bars) are calculated with B3LYP/def2-QZVP/D3.

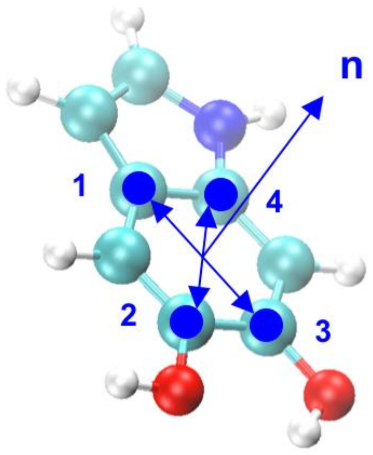

Figure S6 | Definition of orientation vector of DHI monomer.

Supplementary Tables

ID	Bonding	Energy	ID	Bonding	Energy	ID	Bonding	Energy
1	4-7′	2.34*	7	2-7′	4.19*	13	4-7′	2.31
2	7-7′	4.96*	8	2-4′	1.95*	14	4-4′	2.49*
3	7-7′	3.20	9	2-2′	0.00	15	2-4′	3.07*
4	4-7′	4.17*	10	2-2′	1.36*	16	2-4′	1.82
5	2-7′	4.19*	11	2-4′	3.06*	17	4-4′	2.19
6	2-7′	2.86*	12	2-7′	2.82	18	4-7′	2.42*

Table S1 | Relative energies and covalent bonding positions of all dimers. The energies in the table are relative energies compared to the most stable dimer (Dimer-9). The unit of energy is kcal/mol and * indicates higher energy conformations. The most stable dimer is made through 2,2′-position, followed by 2,4′-position (+1.82 kcal/mol), 4,4′-position (+2.19 kcal/mol), 4,7′-position (+2.31 kcal/mol), 2,7′-position (+2.82 kcal/mol), and 7,7′-position (+3.20 kcal/mol).

ID	Energy	ID	Energy	ID	Energy	ID	Energy	ID	Energy	ID	Energy
1	3.37	37	7.97	73	6.55	109	4.70	145	3.72	181	4.74
2	4.53	38	8.58	74	5.24	110	5.30	146	3.93	182	2.64
3	6.55	39	8.16	75	7.01	111	3.69	147	1.09	183	3.64
4	5.76	40	5.73	76	5.62	112	2.18	148	2.55	184	4.38
5	4.82	41	5.03	77	2.91	113	2.71	149	3.65	185	6.15
6	3.51	42	7.15	78	4.43	114	4.06	150	3.84	186	4.40
7	4.15	43	4.42	79	4.82	115	4.41	151	3.54	187	3.13
8	2.74	44	3.06	80	6.30	116	2.16	152	3.75	188	3.26
9	0.78	45	4.88	81	5.32	117	2.64	153	4.07	189	2.51
10	2.02	46	6.24	82	3.76	118	2.82	154	2.41	190	2.73
11	5.17	47	5.02	83	4.88	119	2.23	155	3.55	191	4.82
12	3.76	48	6.03	84	7.23	120	3.55	156	5.77	192	4.07
13	4.68	49	7.02	85	4.85	121	6.96	157	3.34	193	6.57
14	6.81	50	6.57	86	4.38	122	6.59	158	3.50	194	5.78
15	9.11	51	7.04	87	2.74	123	5.37	159	2.20	195	4.96
16	6.92	52	6.61	88	2.99	124	4.08	160	1.03	196	3.81
17	5.84	53	3.71	89	1.30	125	4.28	161	4.52	197	3.50
18	5.22	54	5.46	90	0.00	126	5.28	162	5.51	198	4.70
19	5.95	55	5.67	91	6.01	127	5.76	163	4.62	199	0.98
20	5.31	56	4.32	92	2.59	128	4.62	164	3.72	200	2.00
21	3.53	57	5.21	93	2.71	129	1.58	165	3.86	201	3.55
22	2.31	58	7.18	94	3.90	130	2.68	166	5.01	202	4.96
23	3.98	59	6.95	95	5.02	131	4.69	167	5.92	203	5.17
24	7.66	60	5.56	96	6.39	132	4.46	168	6.44	204	4.10
25	6.56	61	6.04	97	1.02	133	5.23	169	5.11	205	8.09
26	8.88	62	4.51	98	2.33	134	3.64	170	3.48	206	5.80
27	8.95	63	2.65	99	0.79	135	2.78	171	4.61	207	5.01
28	6.37	64	5.28	100	0.99	136	4.08	172	4.64	208	6.28
29	7.14	65	4.35	101	1.54	137	3.77	173	3.91	209	5.98
30	5.58	66	4.81	102	0.13	138	3.69	174	6.01	210	5.78
31	4.02	67	5.42	103	3.86	139	6.44	175	6.46	211	3.26
32	4.77	68	5.20	104	2.22	140	4.40	176	4.14	212	3.23
33	6.82	69	5.30	105	2.91	141	3.65	177	5.53	213	3.66
34	7.67	70	7.83	106	1.43	142	1.91	178	7.43	214	5.58
35	7.05	71	4.81	107	1.08	143	4.20	179	6.94	215	7.09
36	5.30	72	4.43	108	4.20	144	5.97	180	6.36	216	6.41

Table S2 | Relative energies of all trimers. The energies in the table are relative energies compared to the most stable trimer (Trimer-90). The unit of energy is kcal/mol.

Supplementary Method

Method used to calculate projection product of DHI oligomer:

1. Locate the coordinates of the four reference atoms (Fig. S6) on each DHI monomer
2. Generate the reference vectors, $Vector\ 13_i$ and $Vector\ 24_i$, for the *i*-th DHI monomer
3. Define the orientation vector, $Vector\ n_i = Vector\ 13_i \times Vector\ 24_i$, for the *i*-th DHI monomer
4. Normalize $Vector\ n_i$ to a unit vector
5. The projection product of a DHI oligomer made of *m* DHI monomers is defined as:

$$Projection\ product = \sum_{i=1}^{m-1} |Vector\ n_i \cdot Vector\ n_{i+1}| / (m-1)$$
